# Supplementary material for: Catalytic Ring-Opening Polymerisation of Cyclic Ethylene Carbonate: Importance of Elementary Steps for Determining Polymer Properties Revealed via DFT-MTD Simulations Validated Using Kinetic Measurements
Source: Polymers (Basel). 2023 Dec 31;16(1):136. doi: 10.3390/polym16010136 (PMC10781105; doi:10.3390/polym16010136)
Supplement: Supplementary file 1 [file polymers-16-00136-s001.zip › polymers-2754400-supplementary.pdf]

**Supporting information for**

# Catalytic Ring-Opening Polymerisation of Cyclic Ethylene Carbonate: Importance of Elementary Steps for Determining Polymer Properties Revealed via DFT-MTD Simulations Validated by Kinetic Measurements

**Daniel Brüggemann[1,2], Martin R. Machat[3,2], Reinhard Schomäcker[1], Mojgan Heshmat[3,*]**

[1] Technische Universität Berlin, Institut für Chemie—Technische Chemie, Straße des 17. Juni 124, D-10623 Berlin, Germany
[2] Covestro Deutschland AG, Kaiser-Wilhelm-Alle 60, D-51373 Leverkusen, Germany
[3] RWTH Aachen Universität, ITMC CAT Catalytic Center, Worringerweg 2, D-52074 Aachen, Germany
* Correspondence: mojgan.heshmat@wur.nl; Tel.: +31317481375

## 1 Metadynamics parameters

```
UNITS LENGTH= Å TIME=ps ENERGY=kcal/mol
PACE=100

d1: DISTANCE ATOMS=atom1,atom2
d2: DISTANCE ATOMS=atom1,atom2

METAD ARG=d1,d2  SIGMA=0.4,0.4 HEIGHT=0.35 LABEL=restraint

UPPER_WALLS  ARG=d1  AT=3.5   KAPPA=250.0  EXP=2  EPS=1  OFFSET=0
LABEL=uwall1
UPPER_WALLS  ARG=d2  AT=2.5   KAPPA=250.0  EXP=2  EPS=1  OFFSET=0
LABEL=uwall2
```

The constraints of the MTD (harmonic walls) are defined such that the walls are at least 1.2 – 2.5 Å longer than the normal bond length in each case.

The accuracy of the calculated free energies is 10E-9, which is the precision applied by “sumhills” command running in Plumed.

**Table S1.** Experimentally measured rate constants corresponding to the **Scheme 4** (for $VO_4^{3-}$ anion as the catalyst) at various temperatures.

| T (K) | $k_1$ (1/s) | $k_2$ (1/s) | $k_2/k_1$ |
|---|---|---|---|
| 415 | 0.0349 | 0.0346 | 0.99 |
| 425 | 0.0524 | 0.0534 | 1.02 |
| 435 | 0.1070 | 0.1157 | 1.08 |
| 445 | 0.2071 | 0.2431 | 1.17 |

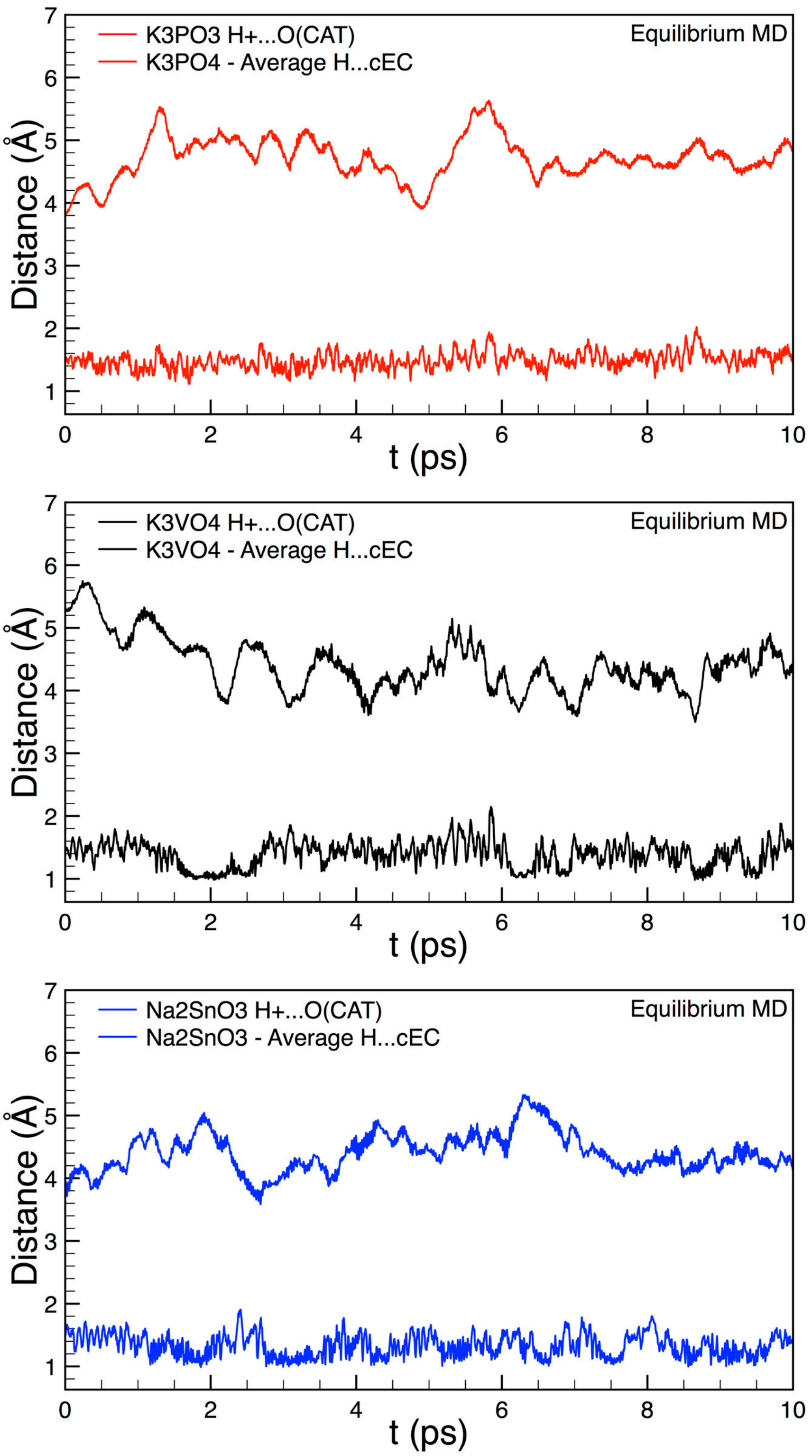


**Figure S1A.** The variation of average H-bond distances between ROH and surrounding cEC monomers (ROH…O(cEC)) in the first solvation shell in comparison to the ROH…O(CAT-anion) alongside an unbiased MD simulations.

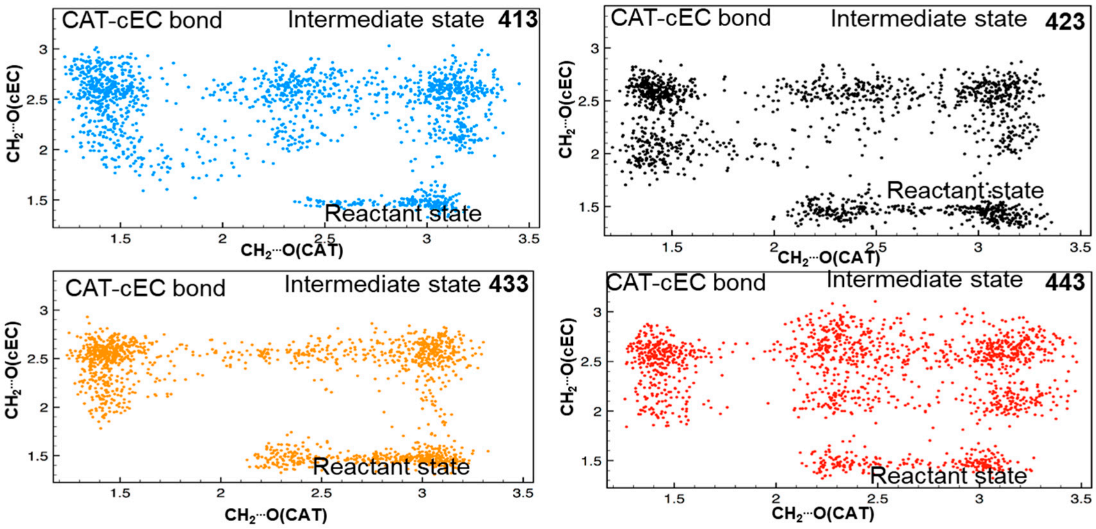


**Figure S1.** Variation of the CVs vs. each other (x,y) for $Na_2SnO_3$ catalyst at four considered temperatures.

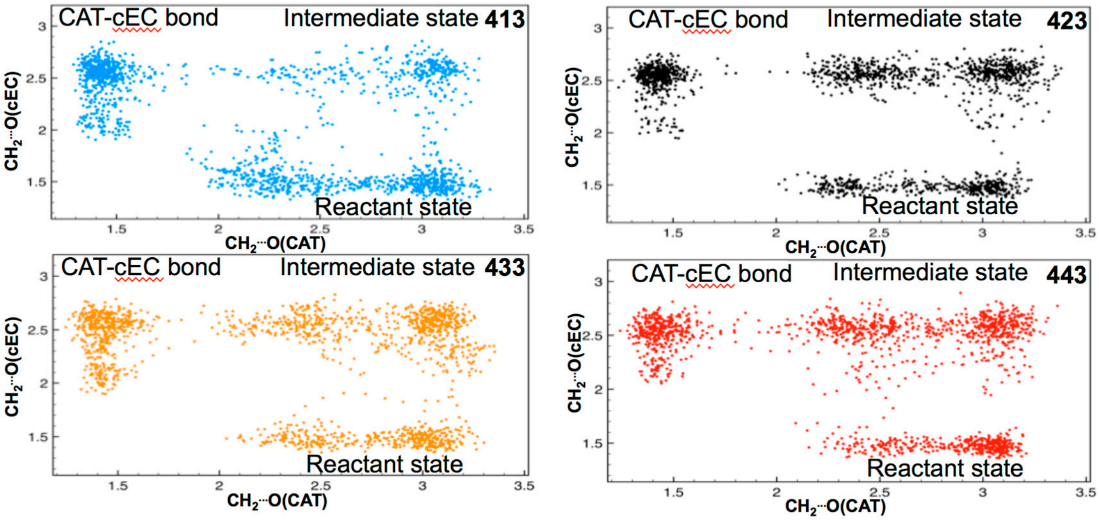


**Figure S2.** Variation of the CVs vs. each other (x,y) for $K_3VO_4$ catalyst at four considered temperatures.

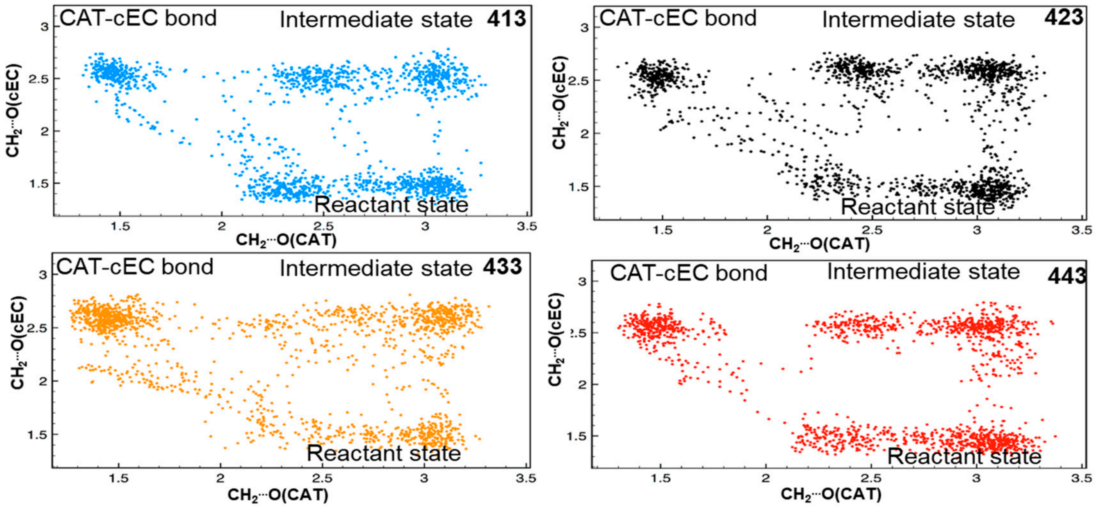


**Figure S3.** Variation of the CVs vs. each other (x,y) for $K_3PO_4$ catalyst at four considered temperatures.

**Variation of the collective variables (CVs) alongside the time evolution of the metadynamics simulations corresponds to Figure 3 in the main text**

CV1 is the O(CAT)···$CH_2$ (nucleophilic attack by the catalyst) distance and CV2 is the O(ethereal)···$CH_2$ (ring cleavage) distance inside the cEC molecule. The distances are shown for four temperatures and for $Na_2SnO_3$, $K_3VO_4$ and $K_3PO_4$ catalysts, **S4 – S6**, respectively. As can be seen in **Figures S4 – S6**, multiple transitions between bond cleavage and/or formation are detected from the CVs behavior.

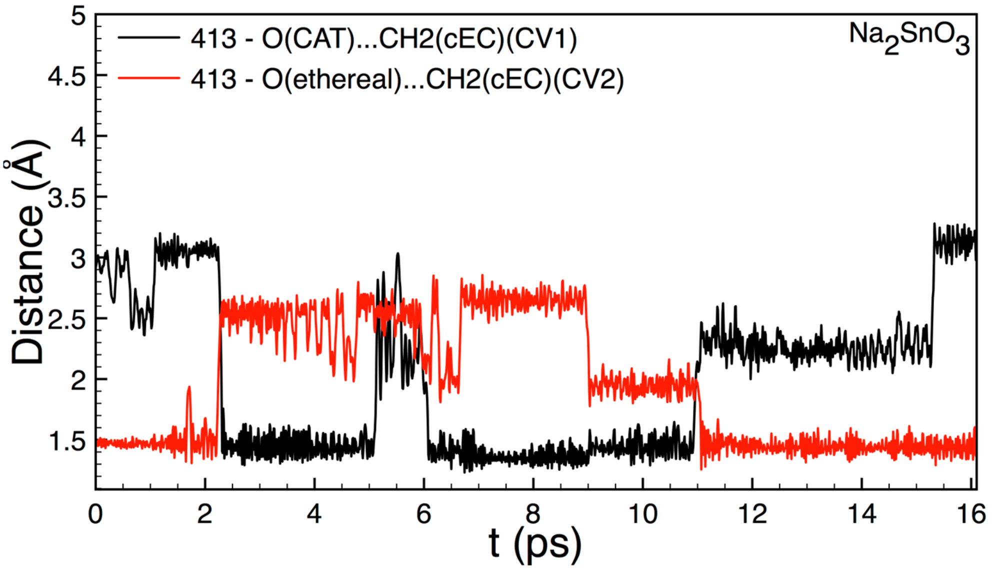

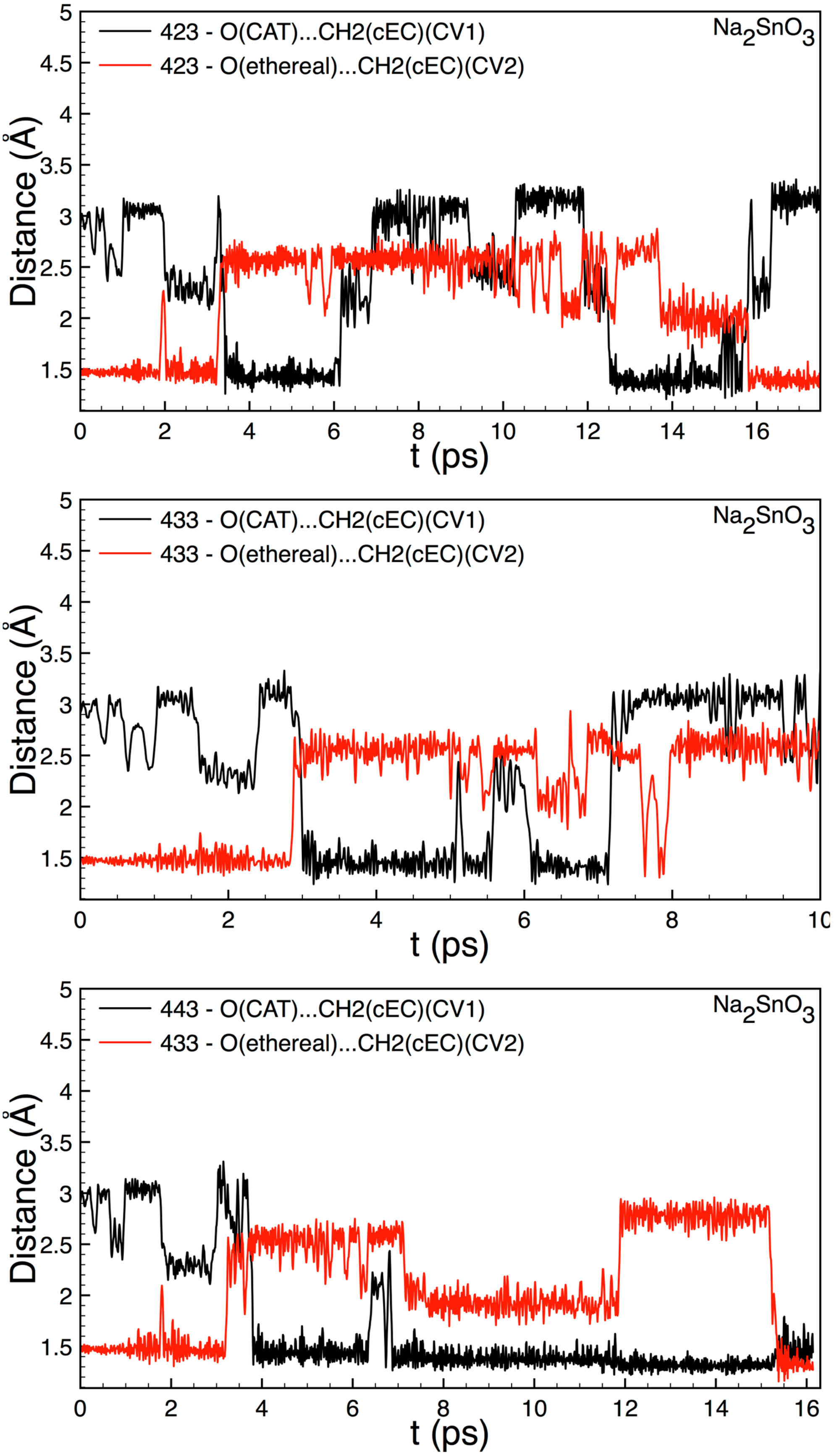


**Figure S4.** The variation of the CVs corresponds to the mechanism shown in **Figure 3** in the main text for $Na_2SnO_3$ catalyst at four considered temperatures.

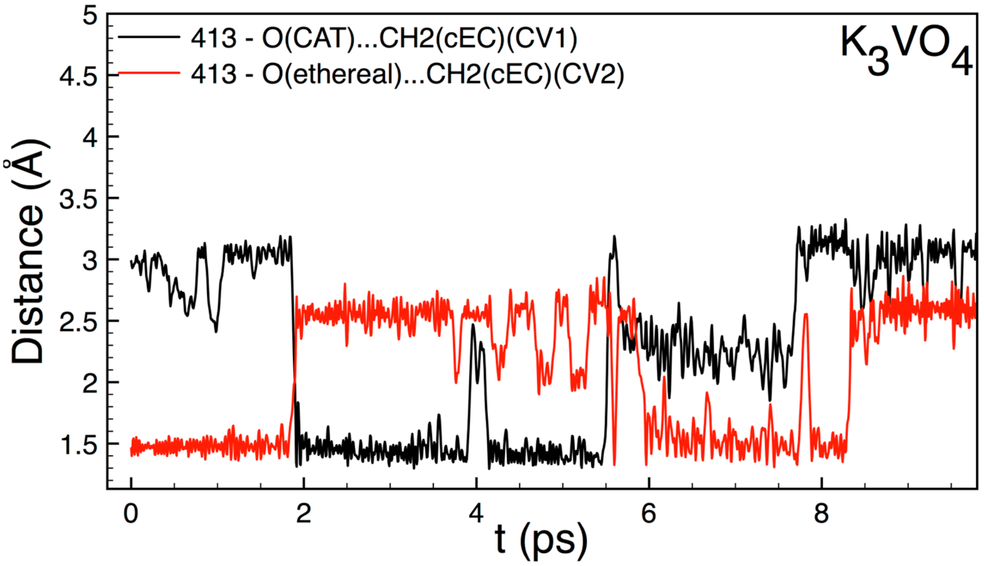
413 - O(CAT)...CH2(cEC)(CV1)
413 - O(ethereal)...CH2(cEC)(CV2)
K3VO4
Distance (Å)
t (ps)

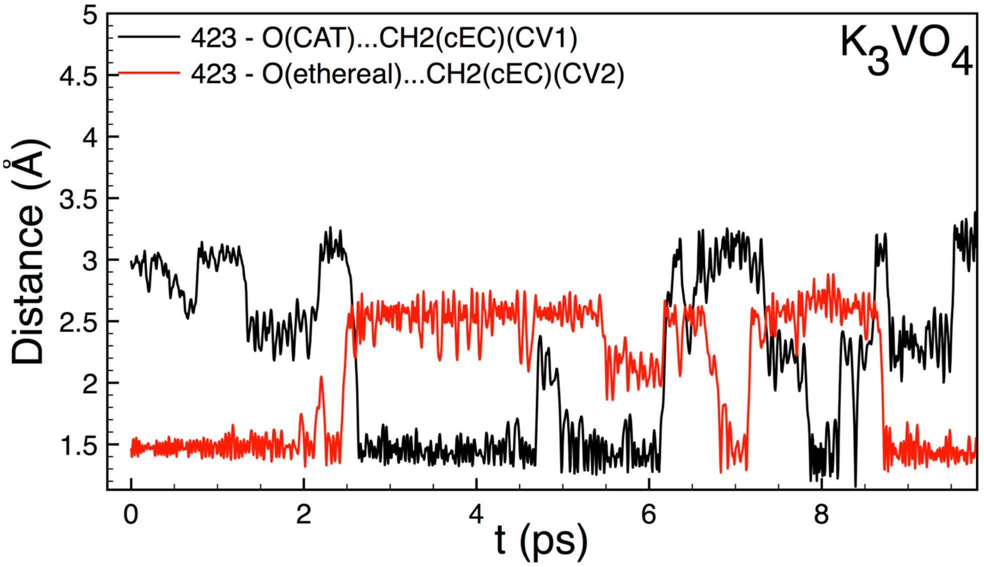
423 - O(CAT)...CH2(cEC)(CV1)
423 - O(ethereal)...CH2(cEC)(CV2)
K3VO4
Distance (Å)
t (ps)

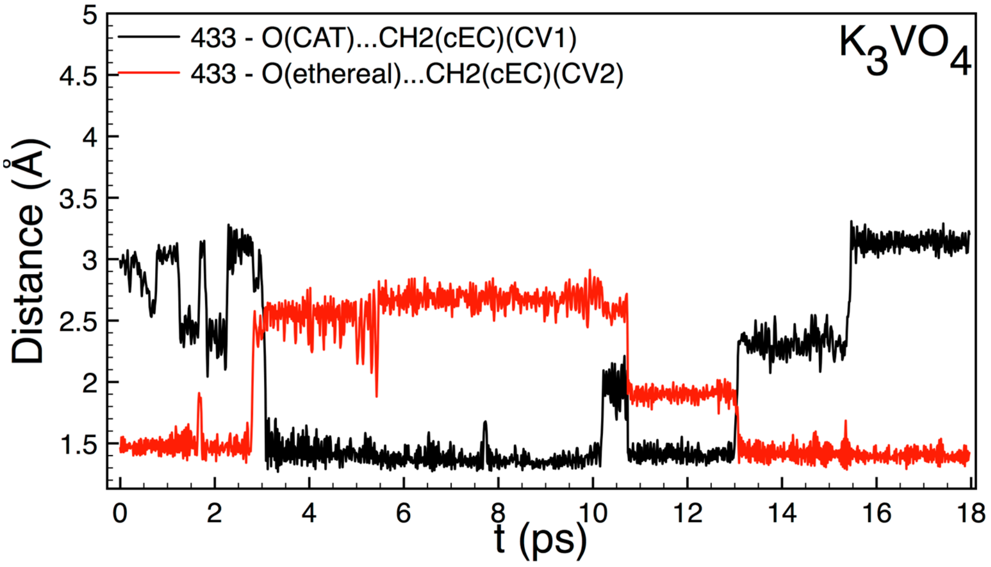
433 - O(CAT)...CH2(cEC)(CV1)
433 - O(ethereal)...CH2(cEC)(CV2)
K3VO4
Distance (Å)
t (ps)

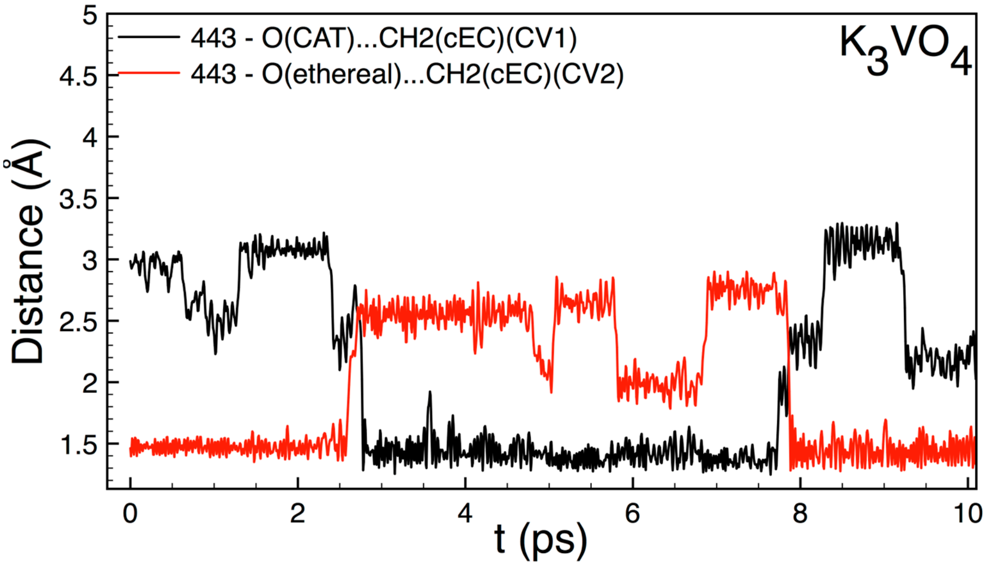


**Figure S5.** The variation of the CVs corresponds to the mechanism shown in **Figure 3** in the main text for $K_3VO_4$ catalyst at four considered temperatures.

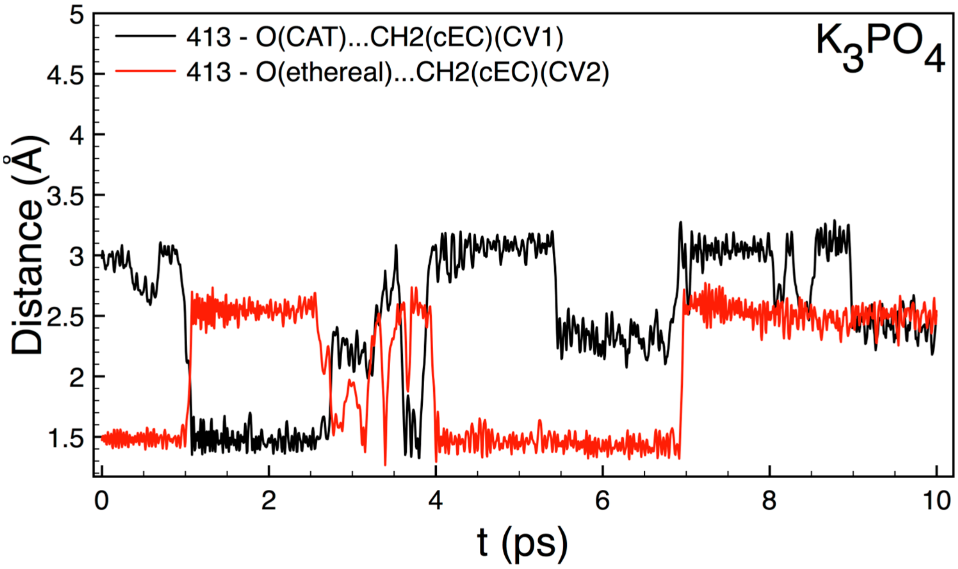


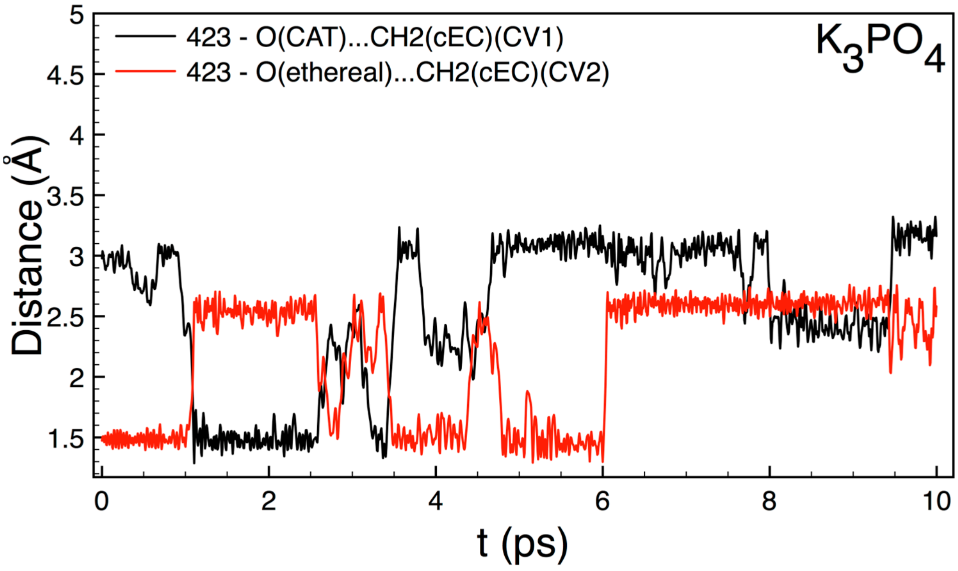

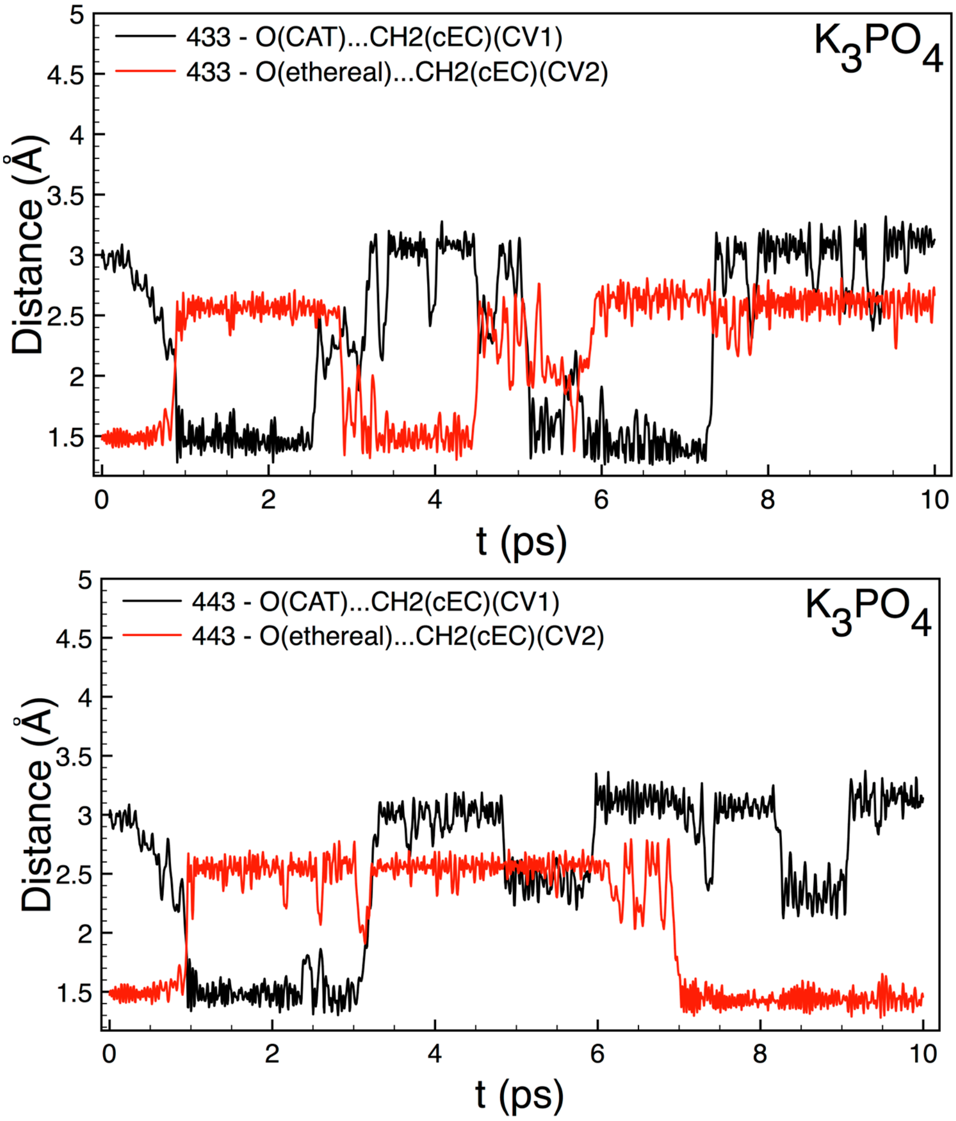


**Figure S6.** The variation of the CVs corresponds to the mechanism shown in **Figure 3** in the main text for $K_3PO_4$ catalyst at four considered temperatures.

**Variation of the collective variables (CVs) alongside the time evolution of the metadynamics simulations corresponds to Figure 9 in the main text for the chain growth mechanism for $K_3VO_4$, $K_3PO_4$ and $Na_2SnO_3$ at four temperatures**

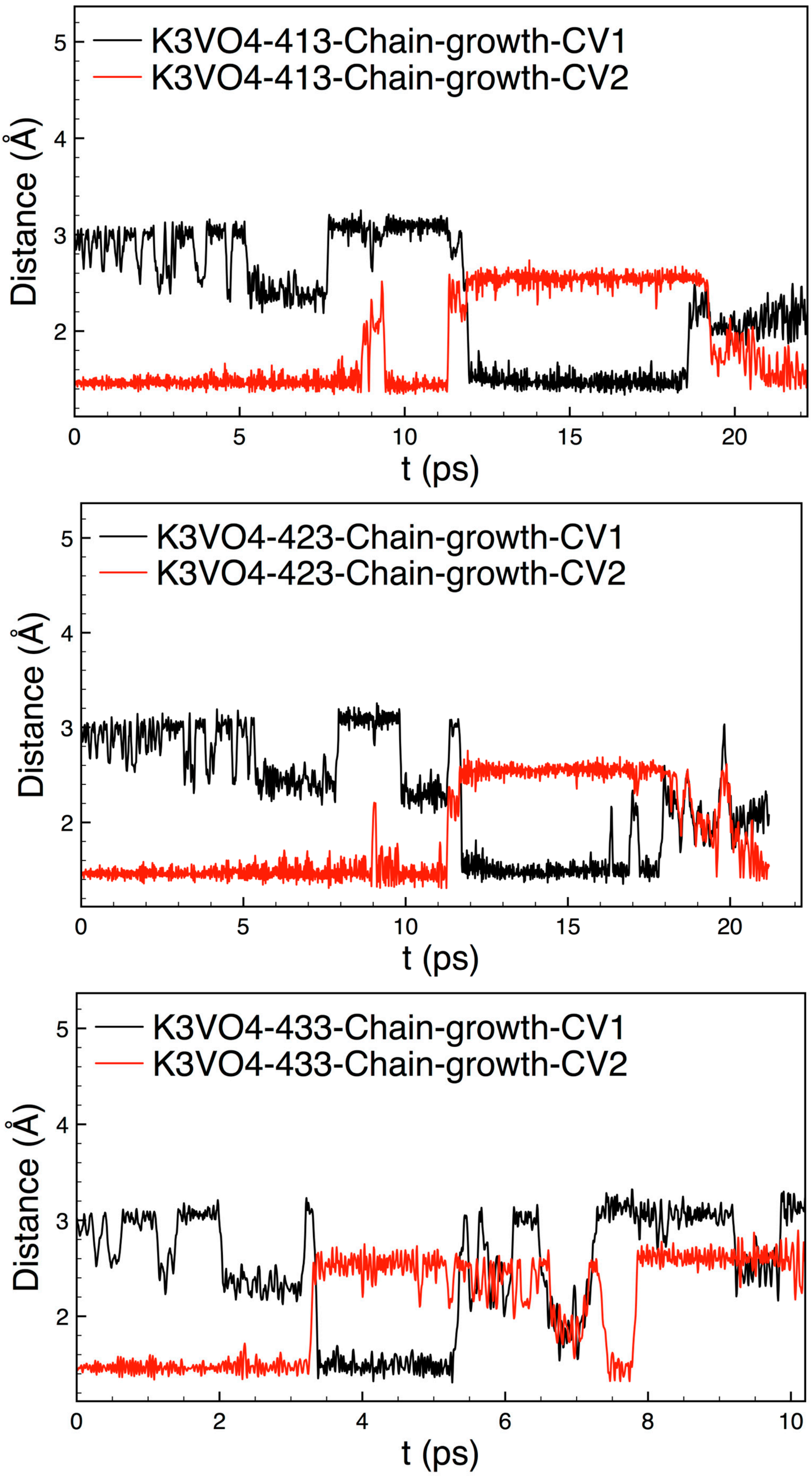
K3VO4-413-Chain-growth-CV1
K3VO4-413-Chain-growth-CV2
Distance (Å)
t (ps)
K3VO4-423-Chain-growth-CV1
K3VO4-423-Chain-growth-CV2
Distance (Å)
t (ps)
K3VO4-433-Chain-growth-CV1
K3VO4-433-Chain-growth-CV2
Distance (Å)
t (ps)

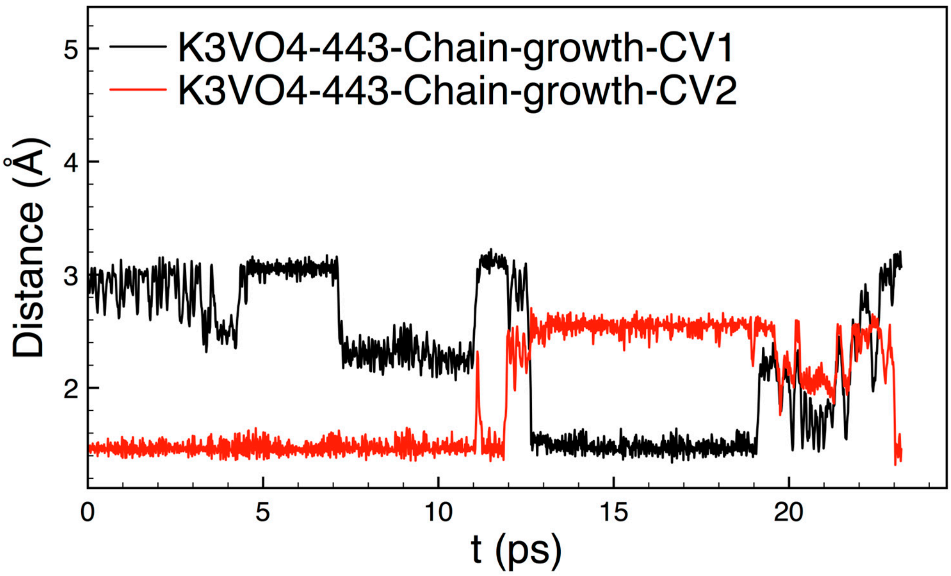


**Figure S7.** The variation of the CVs corresponds to the mechanism shown in **Figure 9** in the main text (chain growth via nucleophilic attack by the $COO^-$ terminal) for $K_3VO_4$ catalyst at four considered temperatures.

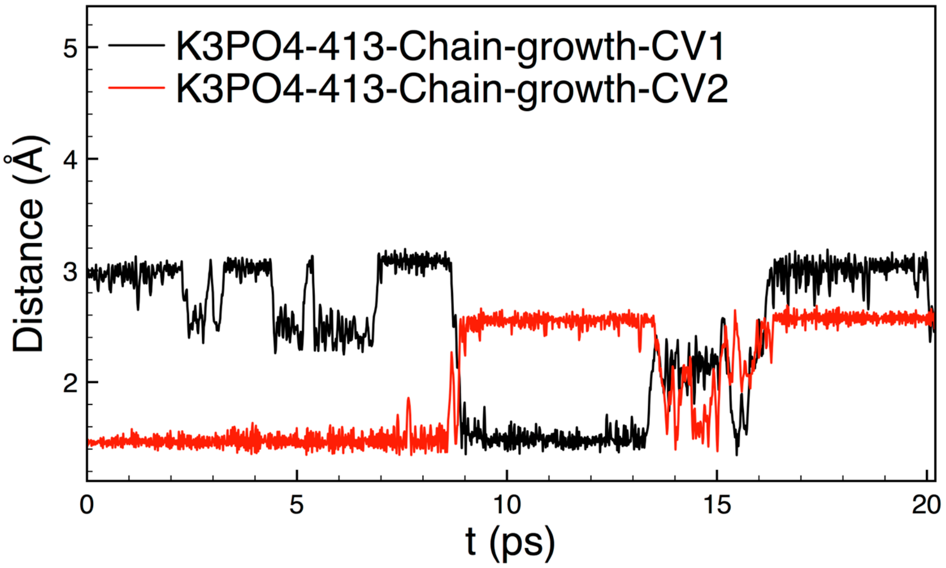

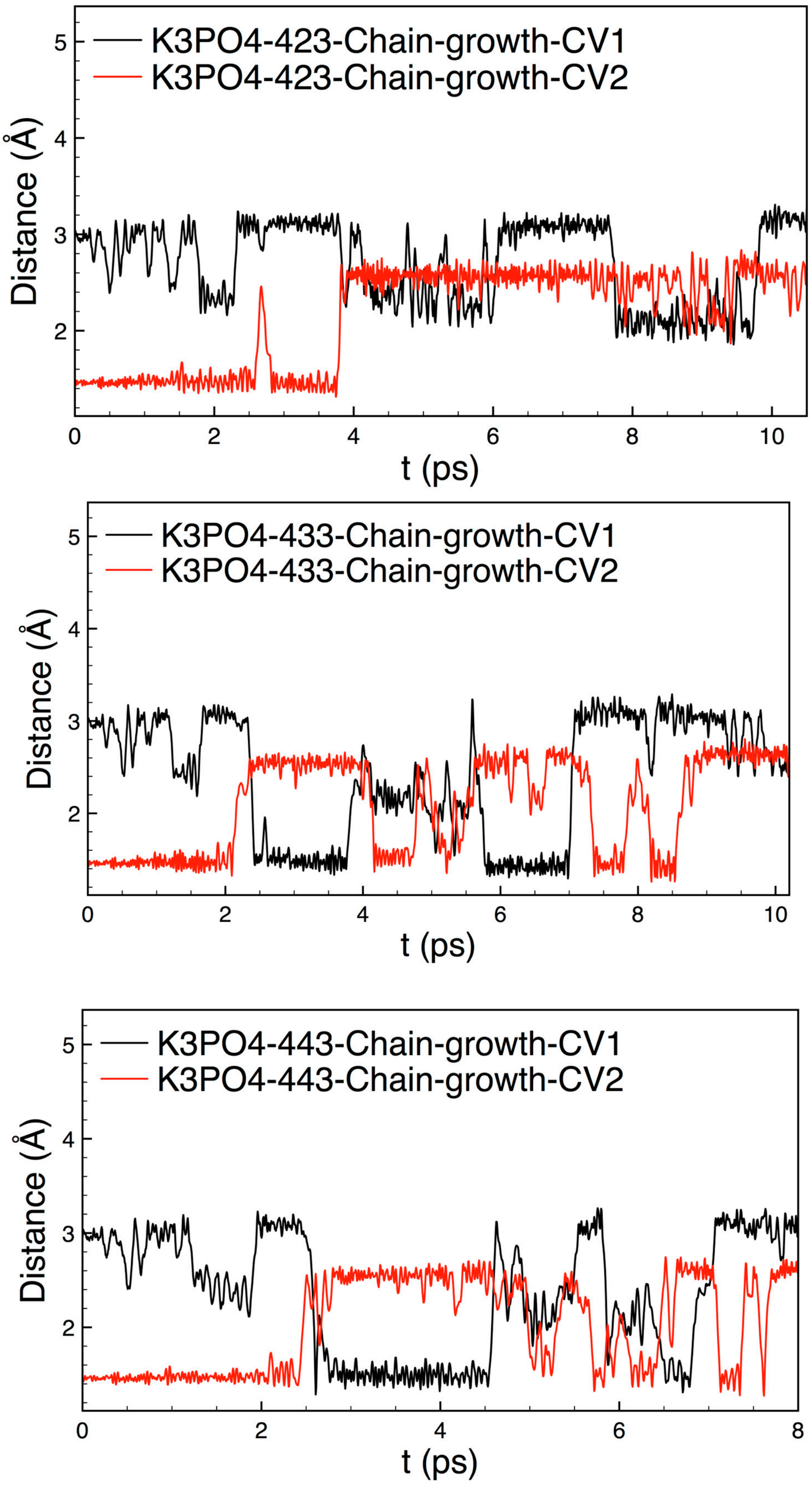


**Figure S8.** The variation of the CVs corresponds to the mechanism shown in **Figure 9** in the main text (chain growth via nucleophilic attack by the $COO^-$ terminal) for $K_3PO_4$ catalyst at four considered temperatures.

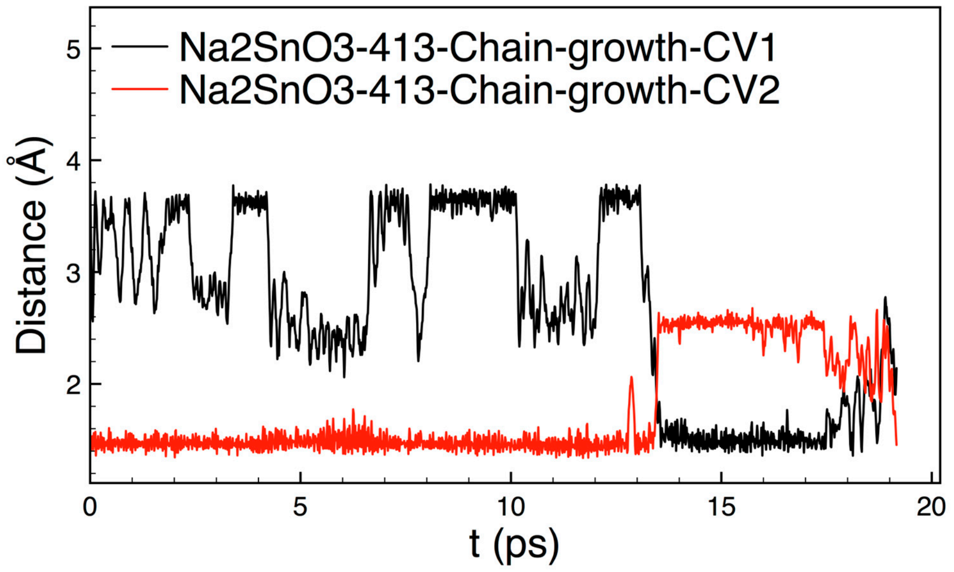
Na2SnO3-413-Chain-growth-CV1
Na2SnO3-413-Chain-growth-CV2
Distance (Å)
t (ps)

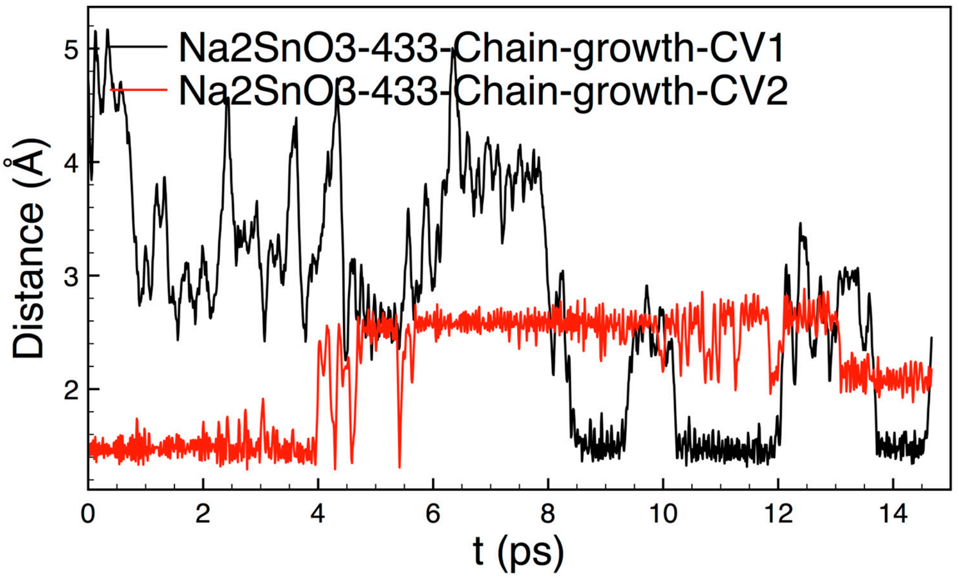
Na2SnO3-433-Chain-growth-CV1
Na2SnO3-433-Chain-growth-CV2
Distance (Å)
t (ps)

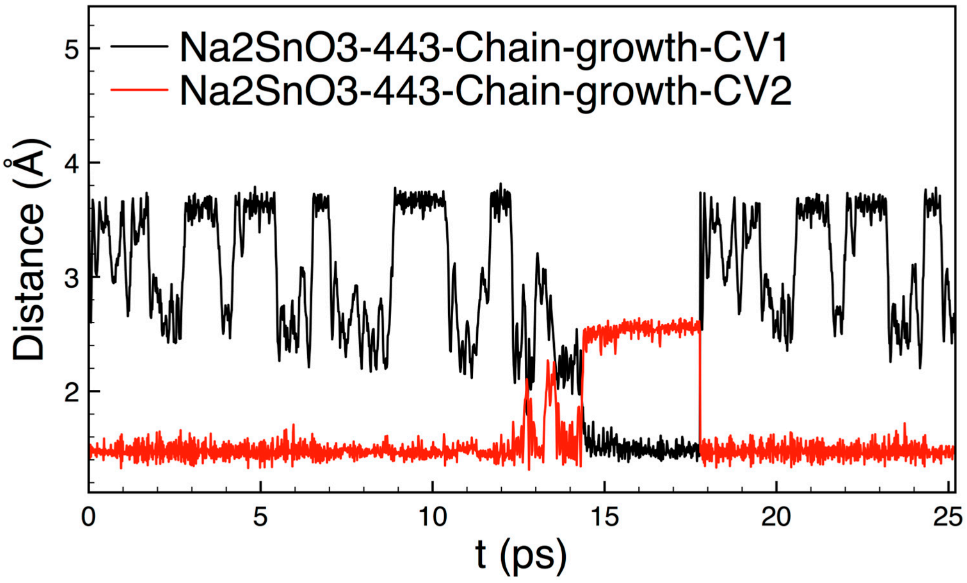
Na2SnO3-443-Chain-growth-CV1
Na2SnO3-443-Chain-growth-CV2
Distance (Å)
t (ps)

**Figure S9.** The variation of the CVs corresponds to the mechanism shown in **Figure 9** in the main text (chain growth via nucleophilic attack by the $COO^-$ terminal) for $Na_2SnO_3$ catalyst at four considered temperatures.

**Variation of the collective variables (CVs) alongside the time evolution of the metadynamics simulations corresponds to Figure 13 in the main text for the nucleophilic attack by the second oxygen of the catalyst anion (Figure S10) and chain transfer mechanisms (Figure S11) for $K_3VO_4$, $K_3PO_4$ and $Na_2SnO_3$ at 423 K.**

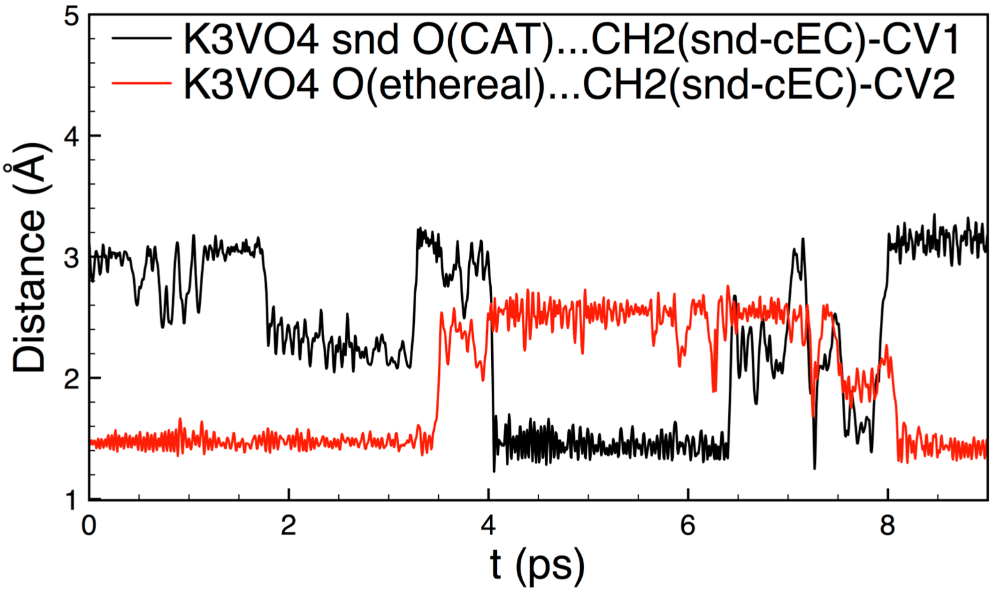


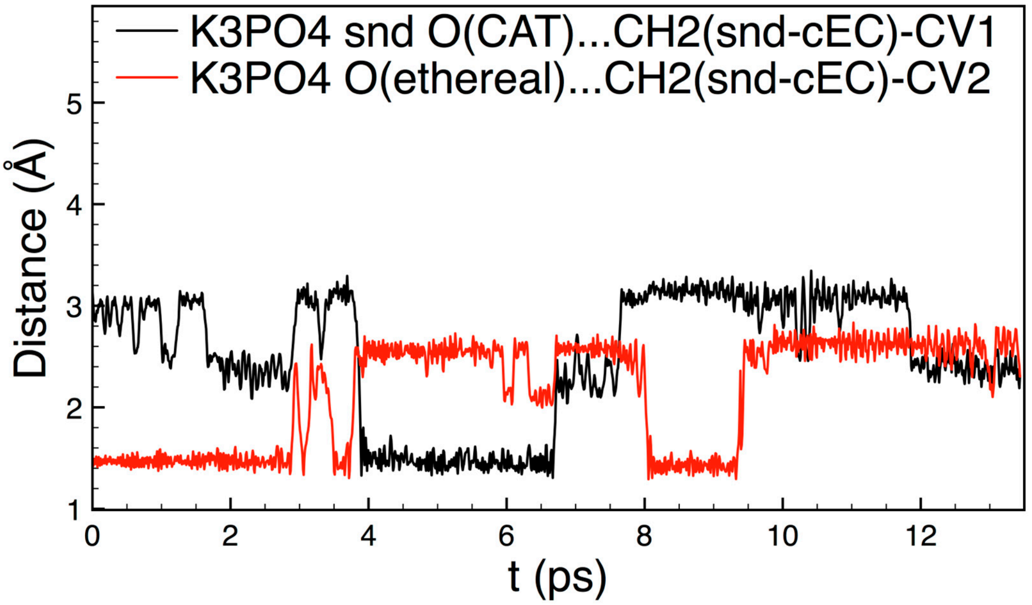

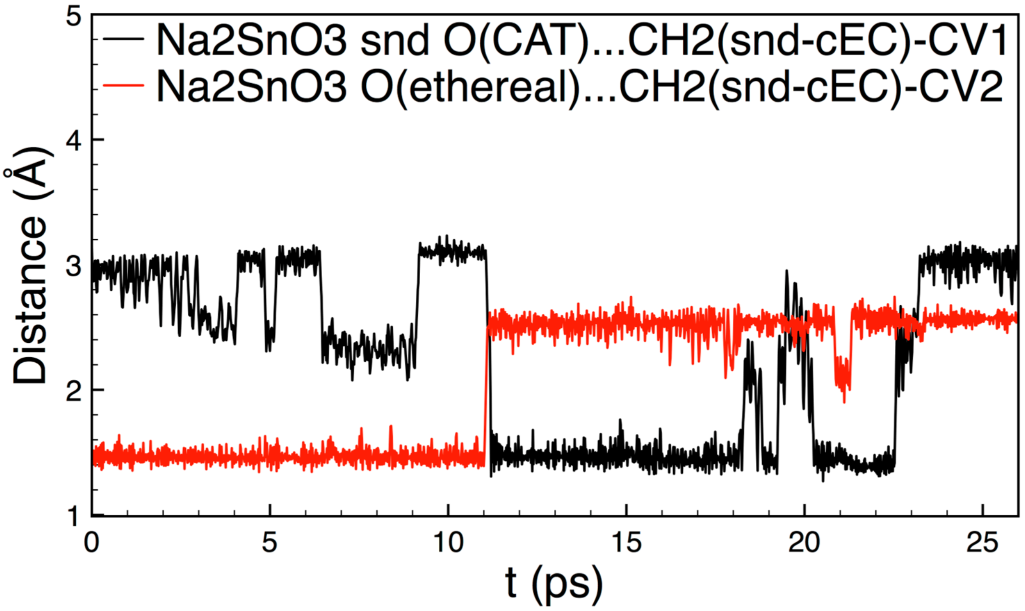


**Figure S10.** The variation of the CVs corresponds to the mechanism shown in **Figure 13** upper part in the main text, i.e., the second nucleophilic attack by the second oxygen of the catalyst for the $K_3VO_4$, $K_3PO_4$ and $Na_2SnO_3$ at 423 K.

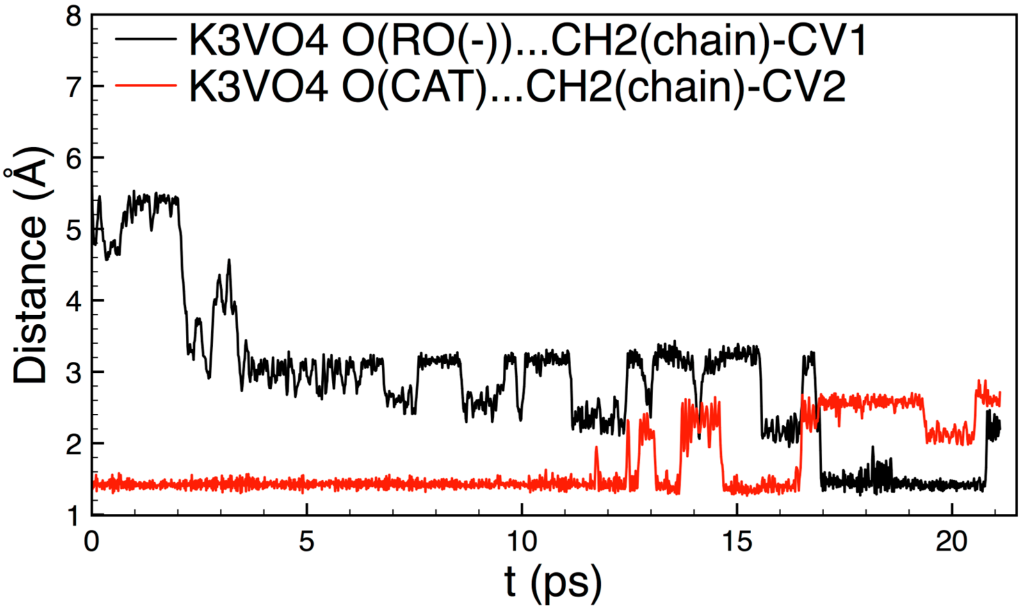

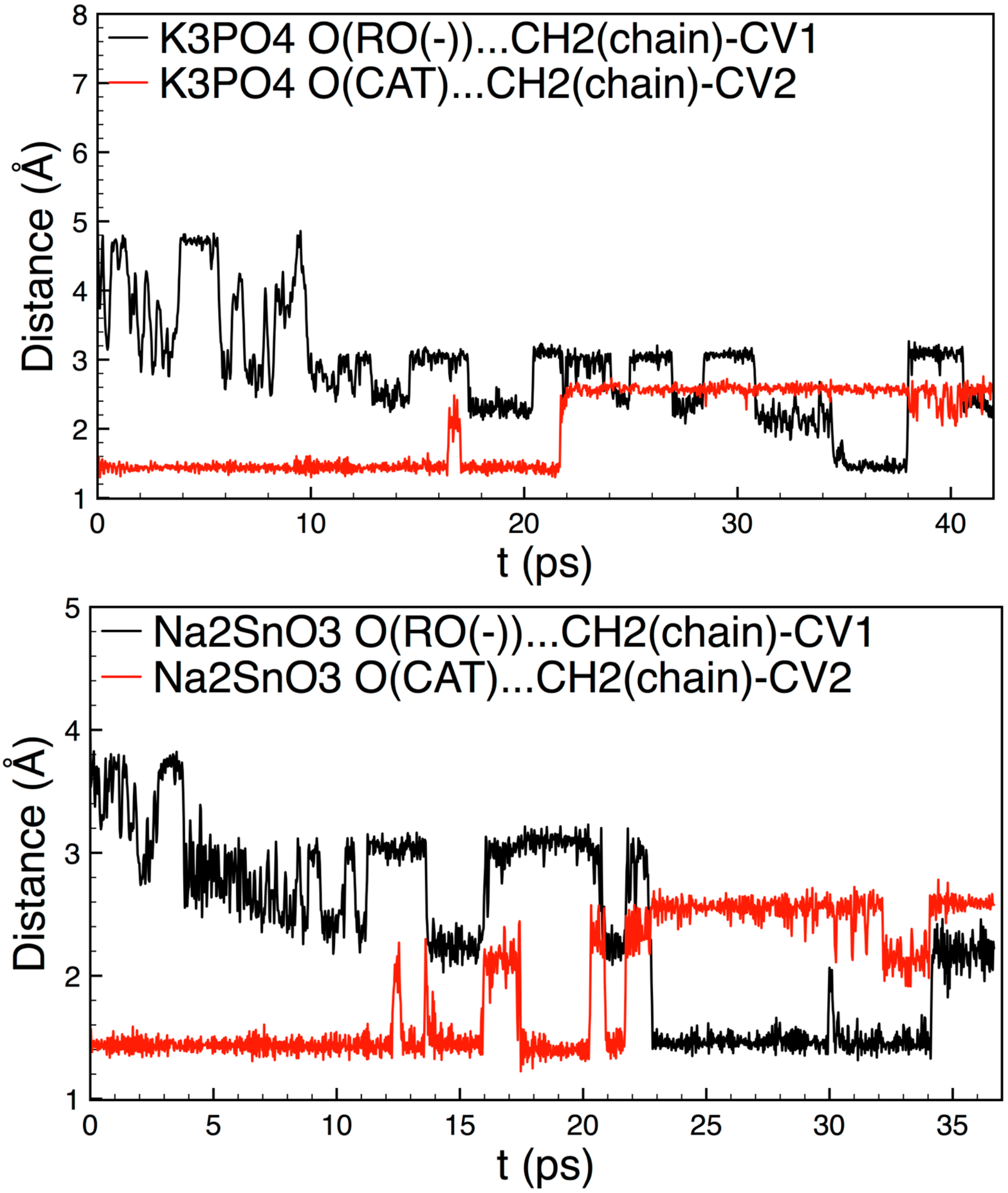


**Figure S11.** The variation of the CVs corresponds to the mechanism shown in **Figure 13** lower part in the main text, i.e., the chain detachment from the catalyst by the nucleophilic attack of another nucleophile in the environment, such as ROH, for the $K_3VO_4$, $K_3PO_4$ and $Na_2SnO_3$ at 423 K.

## 2 Experimental analysis on the kinetics of $CO_2$ release alongside the polymerization of cEC

The reaction rate equations **Error! Reference source not found.-Error! Reference source not found.** reflect the reaction rate for the individual partial reactions in the reaction network. So that the entire system can now be described quantitatively, the rate constants k must first be determined. This is done by numerical simulation of the system with an adjustment of the rate constants to the measured data via the Software Berkeley Madonna. Since in this reaction the consumption of cEC is due to the competitive

reactions between the incorporation or release of $CO_2$ and thus the competition of PECn (linear $CO_2$ ethylene polymer) against PEG (polyethylene glycol), the rate constants k1 and k2 can be described by their sum k', which is adjusted in the simulation in the first step of the evaluation. This sum of the rate constants k' is connected to the individual rate constants $k'_1$ and $k'_2$ via the selectivity (equations (S1), (S2) and (S3)). The selectivity itself can be determined by analysing the products. Since cyclic ethylene carbonate consists of one EO and one $CO_2$ unit each, a $CO_2$ content in the product of maximum 50% can be achieved, assuming that no further $CO_2$ from the atmosphere is incorporated. It is therefore valid that the selectivity S can be expressed according to equation (S1).

$$S = \frac{CO_2 - Content}{0,5} \tag{S1}$$

In order to additionally consider the effect of the catalyst concentration and also the starter concentration in terms of reaction kinetics and to determine their reaction orders n, these concentrations can be included in the rate constants $k_1$ and $k_2$ according to equations (S4) and (S5).

$$k'_1 = S \cdot k' \tag{S2}$$

$$k'_2 = (1 - S) \cdot k' \tag{S3}$$

$$k_1 = k'_1 \cdot c_{starter}^n \cdot c_{cat}^m \tag{S4}$$

$$k_2 = k'_2 \cdot c_{starter}^n \cdot c_{cat}^m \tag{S5}$$

The absorbances of the components in the reaction mixture are determined with time resolution in the experiments. These are related to the concentrations via Bouguer-Lambert-Beer's law (10). Since the measurements were made using an ATR spectrometer, the layer thickness d can be regarded here as λ/2 constant [1] and calculated into the extinction coefficients. Equation (10) now results.

$$EXT_x = c_x \cdot \varepsilon \tag{S6}$$

Since the concentrations of the individual components in the reaction are measured, only the dependence of the individual concentrations on the reaction rate equations **Error! Reference source not found.-Error! Reference source not found.** is needed for the simulation of the temporal change of the system. This can be made possible by differential equations, (S7)-(S10).

$$\frac{dc_{cEC}}{dt} = -r_1 - r_2 \tag{S7}$$

$$\frac{dc_{PECn}}{dt} = r_1 - r_3 - r_4 \tag{S8}$$

$$\frac{dc_{CO_2}}{dt} = r_2 + r_3 + r_4 \tag{S9}$$

$$\frac{dc_{PEG}}{dt} = r_2 + r_4 \tag{S10}$$

$$\frac{dc_{EO}}{dt} = r_3 \tag{S11}$$

With the help of this system of equations, it is now possible to adapt the simulated values to the experimentally determined values by adjusting the rate constants during the simulation. For this purpose, equation (S6) is used to convert the calculated concentration into extinctions for adaptation. The rate constants determined in this way can be used with the help of the Arrhenius equation (S12) or its linearisation to determine the activation energies of the partial steps. The results of these simulations are recorded in tables S2, S3 and S6, the simulation data and NMR data see Supporting Information.

$$k(t) = k_{\infty} \cdot e^{\left(-\frac{E_A}{R \cdot T}\right)} \tag{S12}$$

## 3 Performing the parameter variation of the kinetic measurements

A 50 mL reactor with a heatable jacket was used for all reactions (see figure 9). The reactor is equipped with a GL25 opening for measurement with an in-situ IR probe and has two inlet taps for operation under inert gas. The reactor was heated by using a Haake F6 thermostat. Silicone oil AP200 was chosen as the operating fluid for the thermostat. The IKA RCT Basic magnetic stirrer was used for mixing.

A Mettler Toledo Reakt IR 15 was used for the in-situ measurements. This is equipped with a Si probe connected to the spectrometer via AgX 9.5 mm x 1.5 mm fibre optics. The measured wavenumber range is from 4000 $cm^{-1}$ to 800 $cm^{-1}$ with a resolution of 4 $cm^{-1}$.

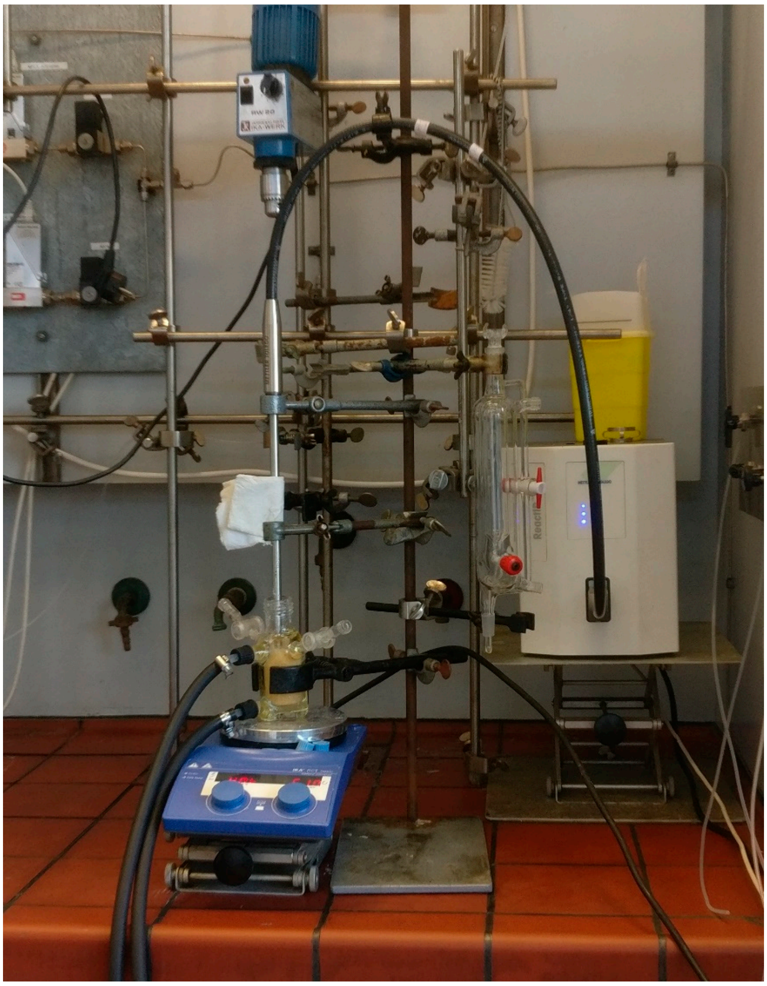

**Figure S12.** Reactor used for the reactions, connected to the in-situ IR spectrometer.

### 3.1 Stannate Catalyst

To determine the activation energy, a series of measurements was taken at different temperatures, but with the same amount of catalyst and starter.

The reactor was brought to the experimental temperature according to Table 3 and the in situ IR probe was supplied with fresh nitrogen (l) for cooling the detector. The blank value of the IR probe was now measured from the empty reactor, in which only the magnetic stirring fish was present. After measuring the blank value, cyclic ethylene carbonate (cEC, 20 g, 1 eq.), which had previously been preheated in a 60 °C water bath, was added to the reactor, the stirrer was set to 600 RPM and the in-situ measurement was started. At least 10 minutes were now waited to obtain a baseline measurement for cEC. Ethylene glycol (10 mol-%, 0.1 eq.) and potassium stannate (1 mol-%, 0.01 eq.), which had been weighed out beforehand, were then added quickly to the reactor.

As the reaction proceeded, the changes in the characteristic bands were monitored: 1800-1760 $cm^{-1}$ [1] for cEC (C-O-C plug-in vibrations, strained system) [1], 1750-1700 $cm^{-1}$ [1] for CO2 (linear non-strained system) [1] and the ether band at 1260 $cm^{-1}$ [1], (these are the expected signals for a nascent poly(ethylene ether carbonate)) [1–5]. Care was taken to ensure that the amount of gas released and its foaming in the reactor did not result in any unwanted discharge from the reactor. The reaction was allowed to run until the band typical of cEC at 1800-1750 $cm^{-1}$ was completely removed. The used experimental parameters for the determination of the activation energy are recorded in **Table S2**.

**Table S2.** Experimental parameters for determining the activation energy with stannate catalyst.

| Experiment | T [°C] | RPM [-] | Cat. [eq.] | Starter [eq.] |
|---|---|---|---|---|
| V041220 | 140 | 600 | 0,01 | 0,1 |
| V031220 | 150 | 600 | 0,01 | 0,1 |
| V041220_160 | 160 | 600 | 0,01 | 0,1 |
| V041220_170 | 170 | 600 | 0,01 | 0,1 |

After determining the activation energy and the effective rate constants, it is useful to investigate the influence of the amount of catalyst on the reaction.

The experiments were carried out according to the experimental procedure described above, varying the amounts of stannate catalyst added (as described in **Table S3**). The reaction was run until the cEC-type band at 1800-1750 $cm^{-1}$ was completely degraded, but for a maximum of 5 hours.

**Table S3.** Experimental parameters to determine the influence of the catalyst quantity on the reaction with stannate catalyst by 150°C.

| Experiment | T [°C] | RPM [-] | Cat. [eq.] | Starter [eq.] |
|---|---|---|---|---|
| V031220 | 150 | 600 | 0,01 | 0,1 |
| V170221_05MP | 150 | 600 | 0,005 | 0,1 |
| V170221_2MP | 150 | 600 | 0,02 | 0,1 |
| V180221_5MP | 150 | 600 | 0,05 | 0,1 |

The products were subsequently examined by $^{1}$H-NMR, whereby deuterated dichloromethane was used for the NMR. In addition, 1 g/L solutions in methanol were analysed by ESI-MS.

### 3.2 Phosphate Catalyst

It was already established in preliminary experiments that potassium phosphate is well suited as a catalyst for the polymerisation of cyclic ethylene carbonate. However, since a problem with incomplete reactions at temperatures below 170°C was observed here, a kinetic investigation of the reaction is more elaborate and the evaluation of the measurements more complex.

A general experimental procedure for the polymerisation of cEC with phosphate as catalyst is to add cyclic ethylene carbonate (1 eq.) preheated to 60°C to a reactor preheated to 150°C. The reaction is started with the addition of cyclic ethylene carbonate (1 eq.). The reaction is started with the addition of finely ground potassium phosphate (0.02 eq.) and continued until no change in the cEC bands can be measured in the IR spectrum, but for a maximum of 5 hours.

To determine the activation energy and the kinetic constants, a series of experiments was measured at different temperatures, but with constant amounts of catalyst and starter. The reactor was brought to operating temperature and the in-situ IR probe was supplied with fresh nitrogen (l). The blank value of the IR probe was now measured from the empty reactor, in which only the magnetic stirring fish was located. After measuring the blank value, cyclic ethylene carbonate (26 g, 1 eq., later abbreviated cEC from here on) was added to the reactor, the stirrer was set to 600 RPM and the in situ measurement was started. It was now waited for at least 10 minutes to obtain a baseline measurement for cEC. Potassium phosphate (0.03 eq.) was now added and finely ground before addition. Care was taken to ensure that the addition took place quickly, as potassium phosphate is hygroscopic. The exact test parameters are listed in **Table S4**. The experiment was stirred for five hours at 600 RPM and the changing bands were examined afterwards (see 3.1 Reaction kinetics). The products were subsequently examined by $^{1}$H- and $^{31}$P-NMR, whereby deuterated dichloromethane was used for the $^{31}$P-NMR. In addition, 1 g/L solutions in methanol were analysed by ESI-MS.

**Table S4**. Experimental parameters for determining the activation energy with phosphate catalyst.

| Experiment | T [°C] | RPM [-] | Cat. [eq.] | Starter [eq.] |
|---|---|---|---|---|
| V220520 | 120 | 600 | 0.03 | 0 |
| V020620 | 130 | 600 | 0.03 | 0 |
| V030620 | 140 | 600 | 0.03 | 0 |
| V040620 | 150 | 600 | 0.03 | 0 |

To investigate the influence of the amount of catalyst on the reaction, a fixed amount of cEC was used at isothermal conditions with variable amounts of catalyst. The reactor was brought to the experimental temperature of 150°C and the in-situ IR probe was supplied with fresh nitrogen (l). The blank value of the IR probe was now measured from the empty reactor, in which only the magnetic stirring fish was located. After measuring the blank value, cyclic ethylene carbonate (25 g, 1 eq.) was added to the reactor and the stirrer was set to 600 RPM.

The reaction mixture was stirred for at least 10 minutes and the measurement with the probe was started. A base spectrum was recorded for a further 10 minutes to establish the starting point of the reaction. Freshly finely ground tribasic potassium phosphate was weighed out in the respective quality according to **Table S5** and added briskly to the reactor. The reaction was observed and changes in the bands recorded. An ex-situ ATR spectrum of a small sample from the reactor was recorded every hour. The reaction was terminated after 5 hours, the mixture was slowly cooled to 88°C and then bottled.

**Table S5.** Experimental parameters to determine the influence of the catalyst quantity on the reaction with phosphate catalyst by 150°C.

| Experiment | T [°C] | RPM [-] | Cat. [eq.] | Starter [eq.] |
|---|---|---|---|---|
| V150720 | 150 | 600 | 0.005 | 0 |
| V160720 | 150 | 600 | 0.01 | 0 |
| V170720 | 150 | 600 | 0.02 | 0 |
| V040620 | 150 | 600 | 0.03 | 0 |
| V280720 | 150 | 600 | 0.05 | 0 |

### 3.3 Vanadate Catalyst

The vanadate catalyst was investigated in the same way as the stannate catalyst (see section 3.1). The reactor was brought to the experimental temperature according to **Table S6** and the in situ IR probe was supplied with fresh nitrogen (l) for cooling the detector. The blank value of the IR probe was now measured from the empty reactor, in which only the magnetic stirring fish was present. After measuring the blank value, cyclic ethylene carbonate (cEC, 20 g, 1 eq.), which had previously been preheated in a 60 °C water bath, was added to the reactor, the stirrer was set to 600 RPM and the in-situ measurement was started. At least 10 minutes were now waited to obtain a baseline measurement for cEC. Ethylene glycol (10 mol-%, 0.1 eq.) and sodium orthovanadate (1 mol-%, 0.01 eq.), which had been weighed out beforehand, were then added quickly to the reactor.

**Table S6.** Experimental parameters for determining the activation energy with vanadate catalyst.

| Experiment | T [°C] | RPM [-] | Cat. [eq.] | Starter [eq.] |
|---|---|---|---|---|
| V270422 | 140 | 600 | 0,01 | 0,1 |
| V080422 | 150 | 600 | 0,01 | 0,1 |
| V250422 | 160 | 600 | 0,01 | 0,1 |
| V060522 | 170 | 600 | 0,01 | 0,1 |

## 4 Adaptation of the kinetic simulation results to the experiments

In this section the results of fitting the kinetic model to the experimental data are presented using the software Berkeley Madonna 8.3.18.

## 4.1 Stannate

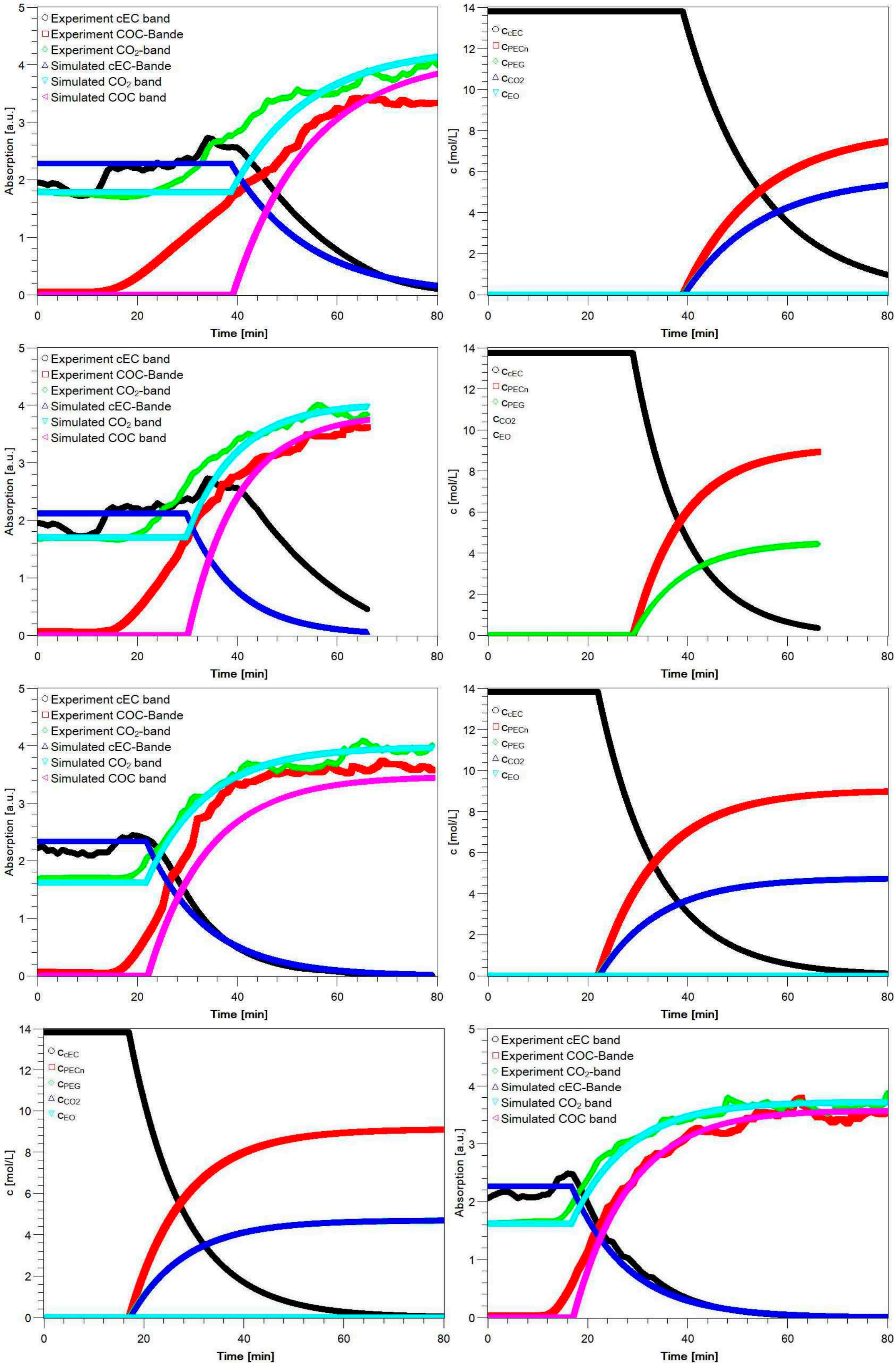


**Figure S13**. Results of fitting the kinetic model to the experimental data for catalyst Stannate. In the temperature range of 140-170°C, stirrer 600 RPM, all parameters see **Table S2**.

## 4.2 Phosphate

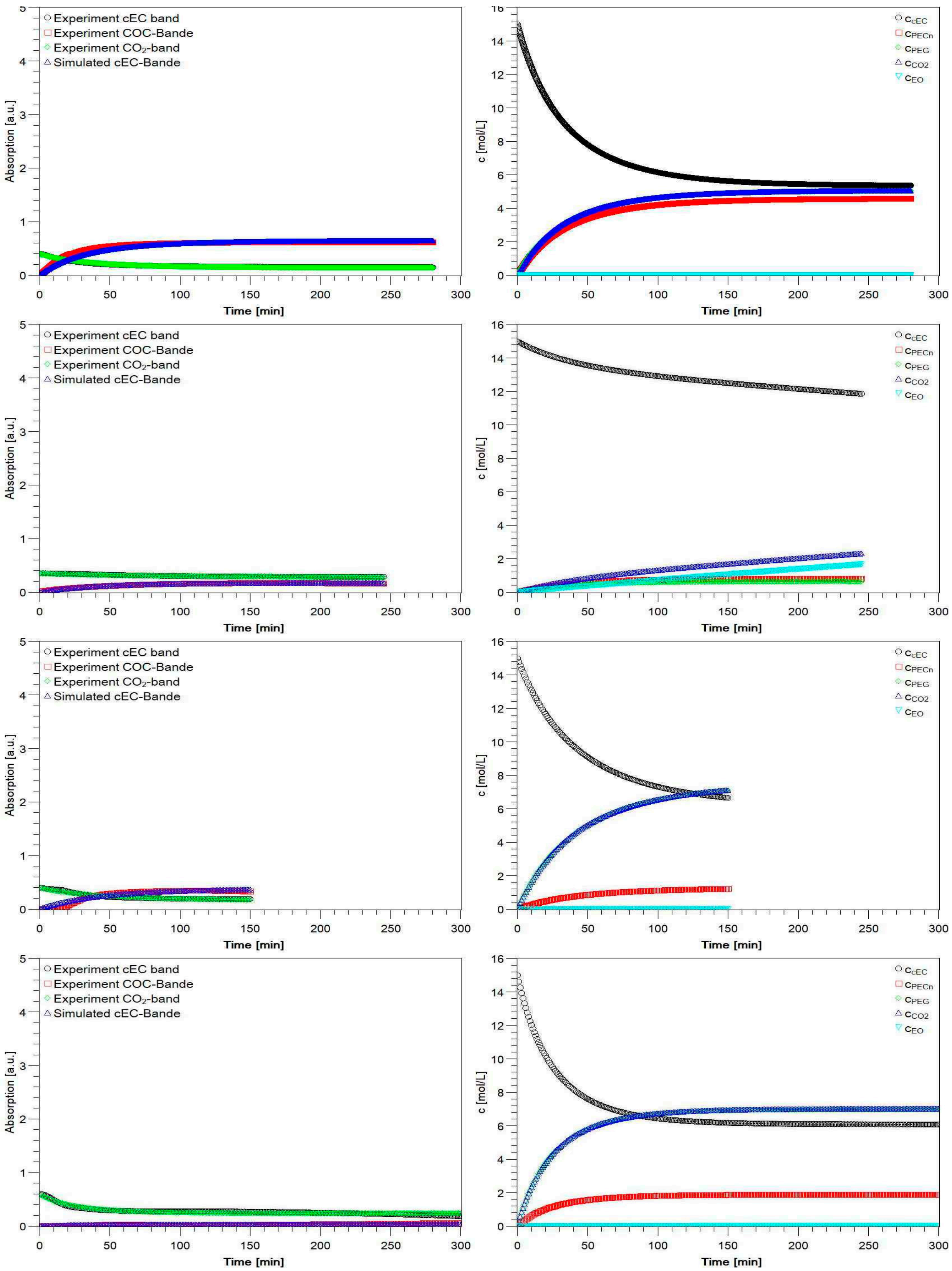


**Figure S14**. Results of fitting the kinetic model to the experimental data for catalyst phosphate. In the temperature range of 120-150, stirrer 600 RPM, all parameters see **Table S4**.

## 4.3 Vanadate

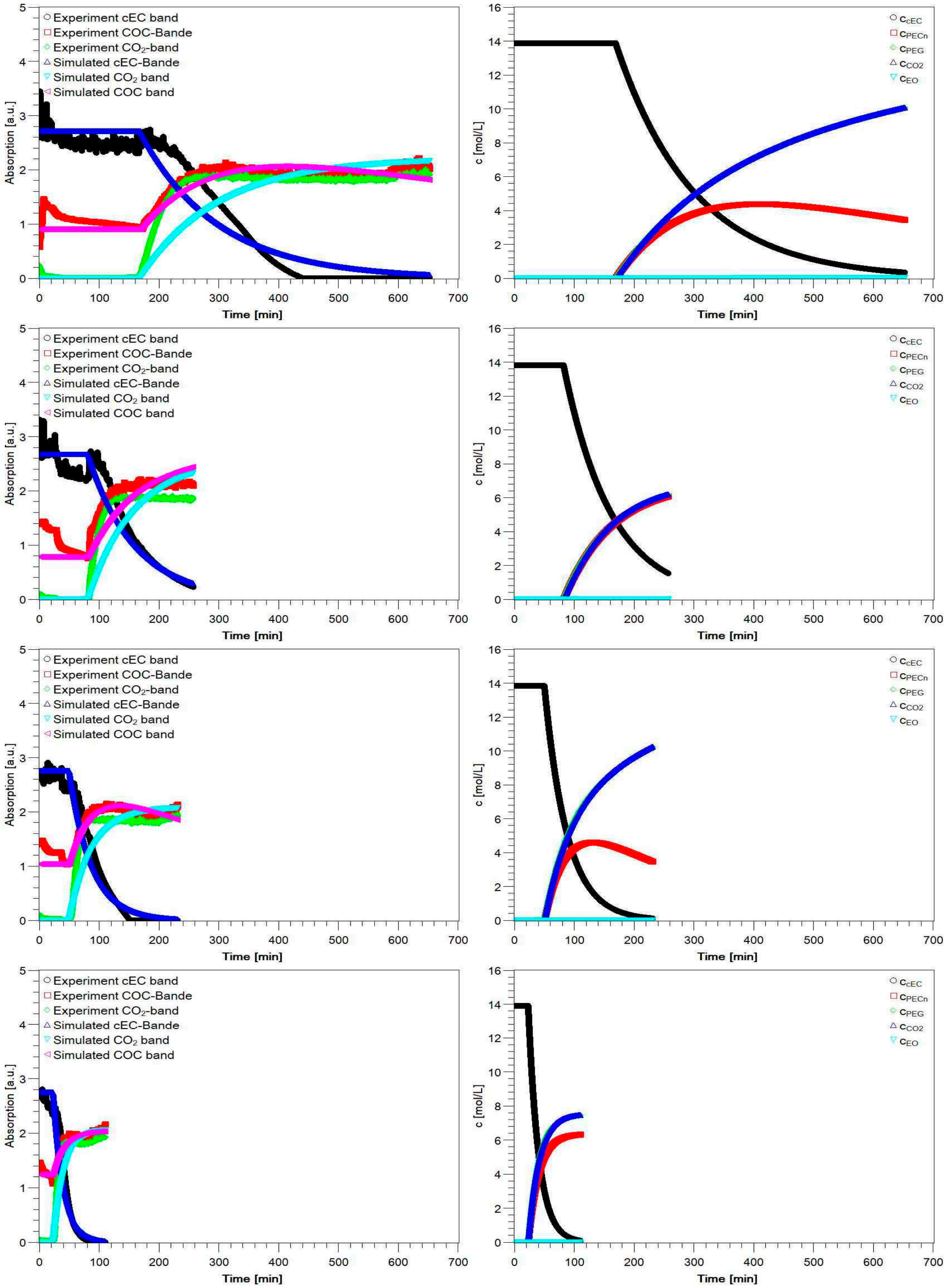


**Figure S15**. Results of fitting the kinetic model to the experimental data for catalyst vanadate. In the temperature range of 140-170, stirrer 600 RPM, all parameters see **Table S6**.

## 5 NMR-Analytik oft he Experiments

In the following section, the $^{1}$H-NMR spectra of the products of the different catalysts are shown.

### 5.1 Stannate

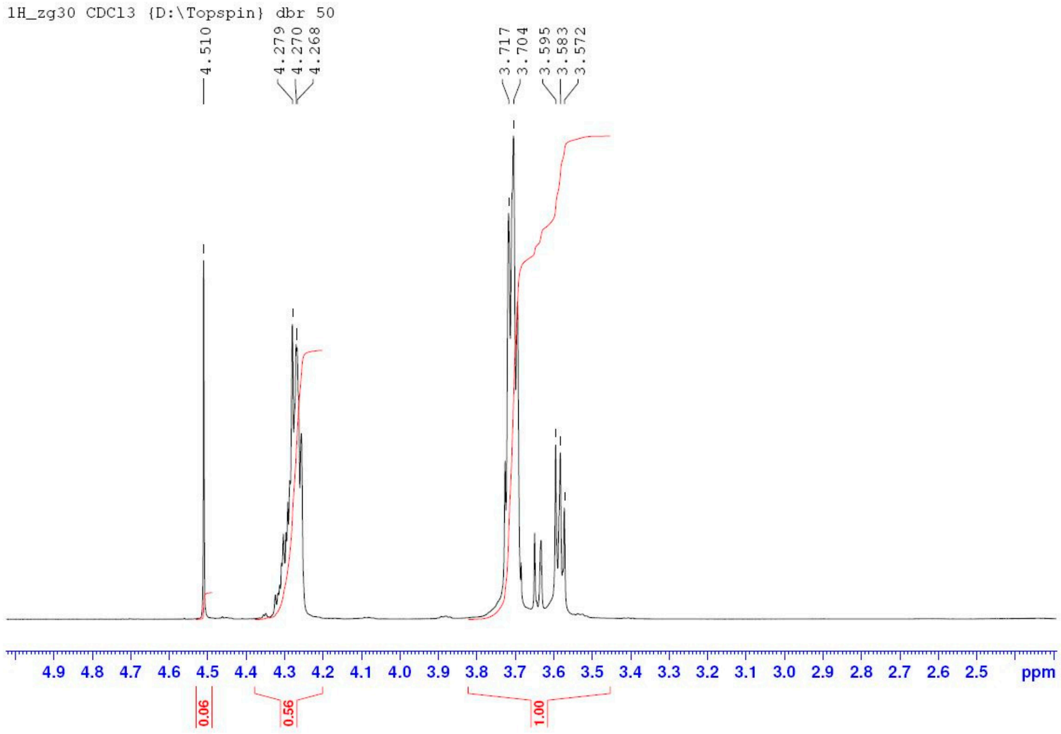


**Figure S16**. $^{1}$H-NMR spectrum in trichloromethane D1 for catalyst stannate at a reaction temperature of 140°C. For reaction conditions see **Table S2**.

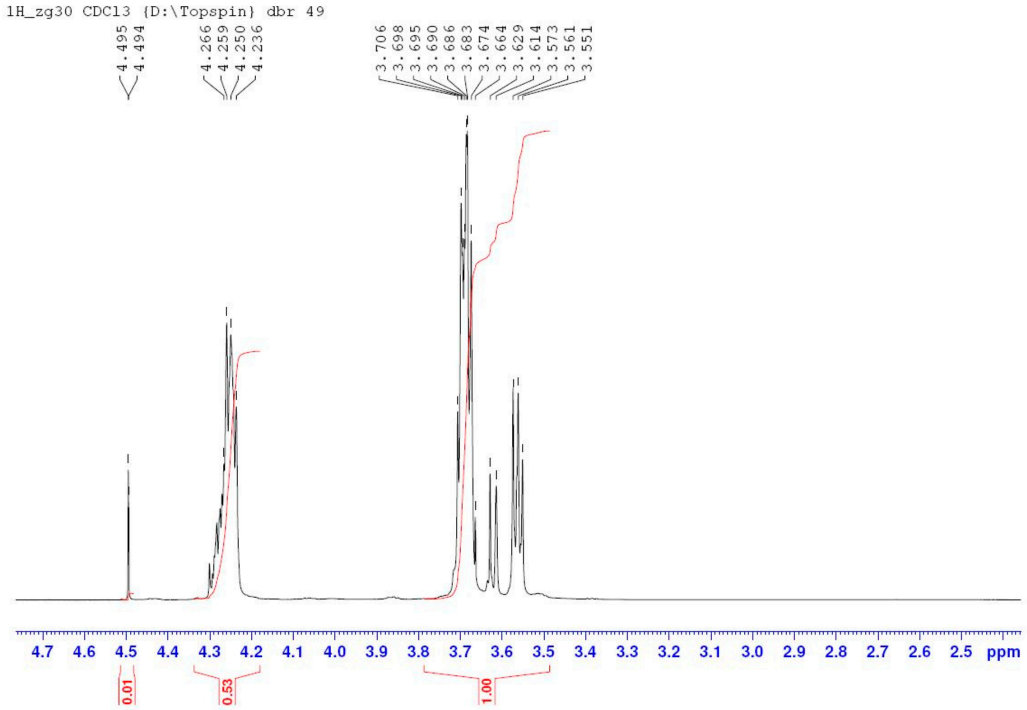


**Figure S17**. $^{1}$H-NMR spectrum in trichloromethane D1 for catalyst stannate at a reaction temperature of 150°C. For reaction conditions see **Table S2**.

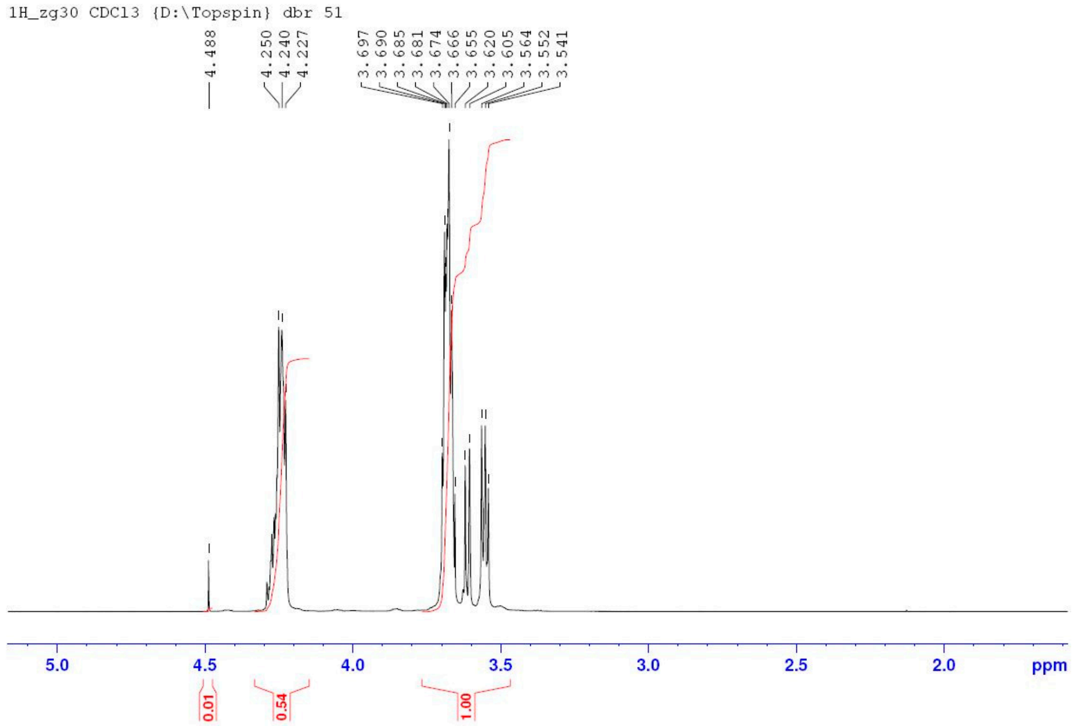


**Figure S18**. $^{1}$H-NMR spectrum in trichloromethane D1 for catalyst stannate at a reaction temperature of 160°C. For reaction conditions see **Table S2**.

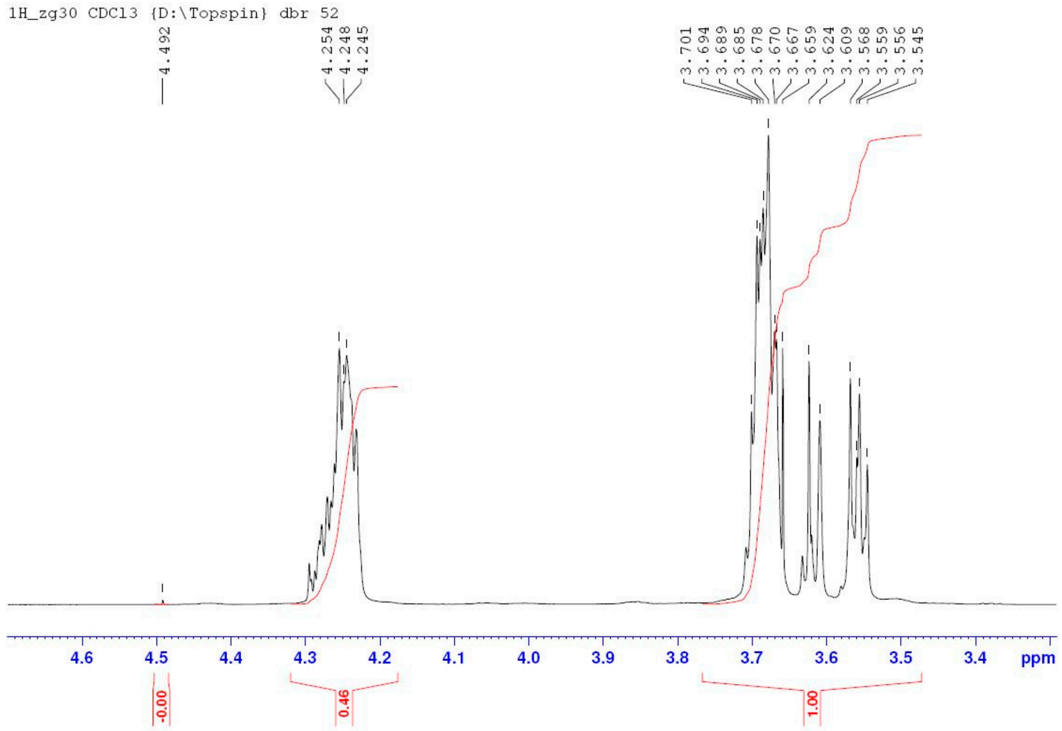


**Figure S19**. $^{1}$H-NMR spectrum in trichloromethane D1 for catalyst stannate at a reaction temperature of 170°C. For reaction conditions see **Table S2**.

## 5.2 Phosphate

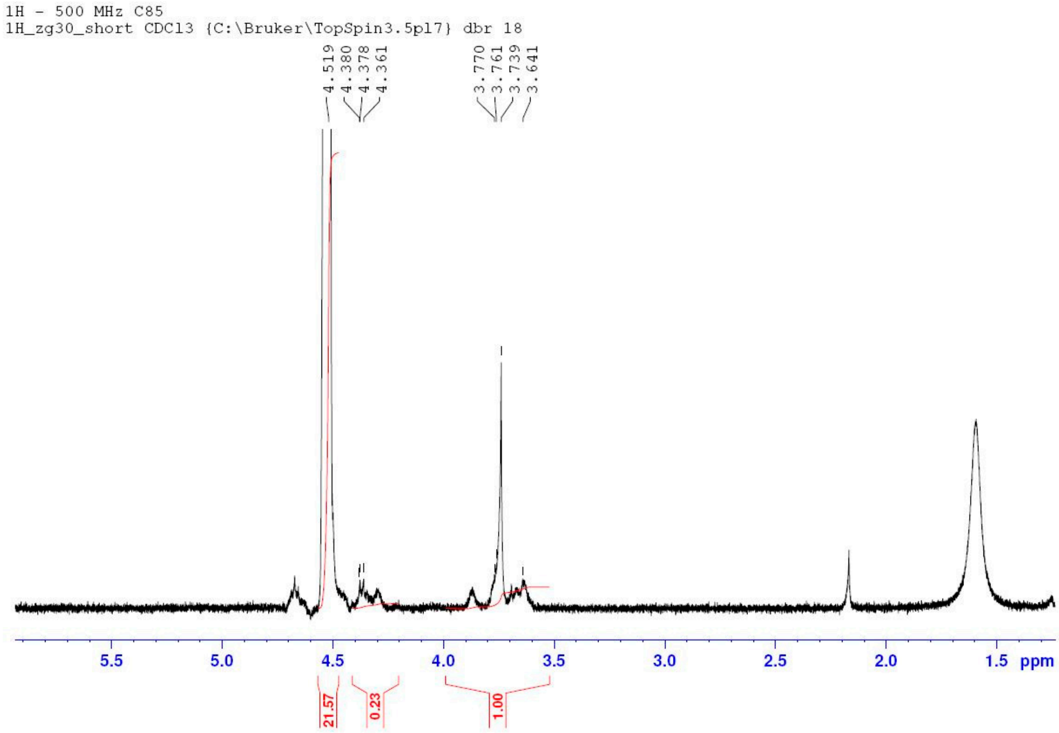


**Figure S20**. $^{1}$H-NMR spectrum in trichloromethane D1 for catalyst phosphate at a reaction temperature of 120°C. For reaction conditions see **Table S4**.

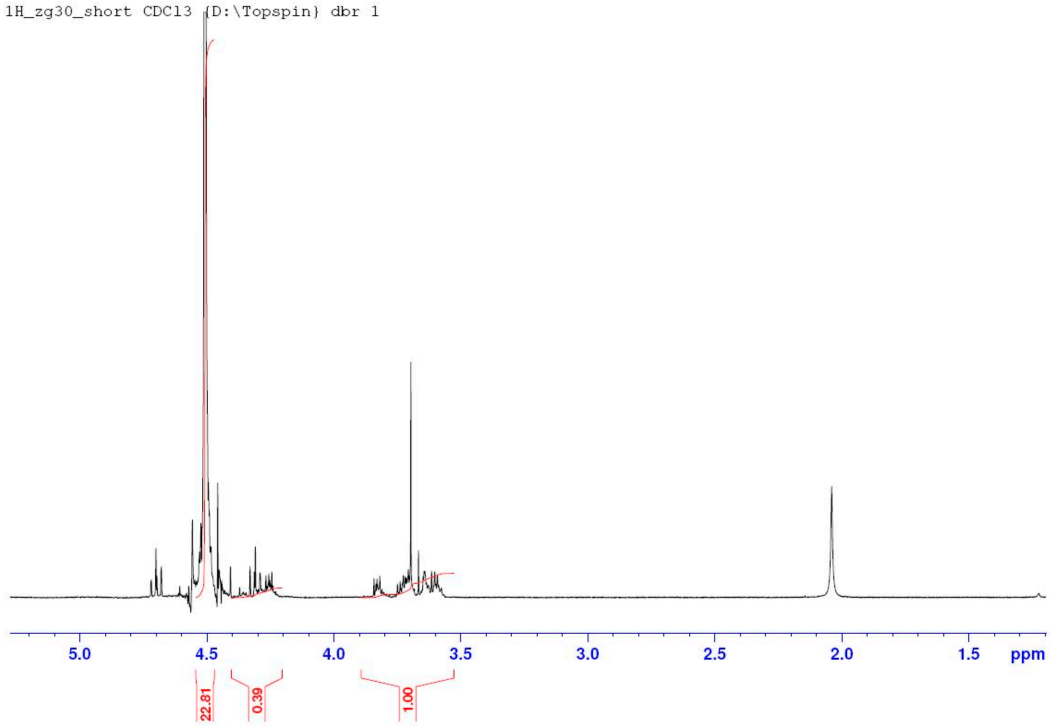


**Figure S21**. $^{1}$H-NMR spectrum in trichloromethane D1 for catalyst phosphate at a reaction temperature of 130°C. For reaction conditions see **Table S4**.

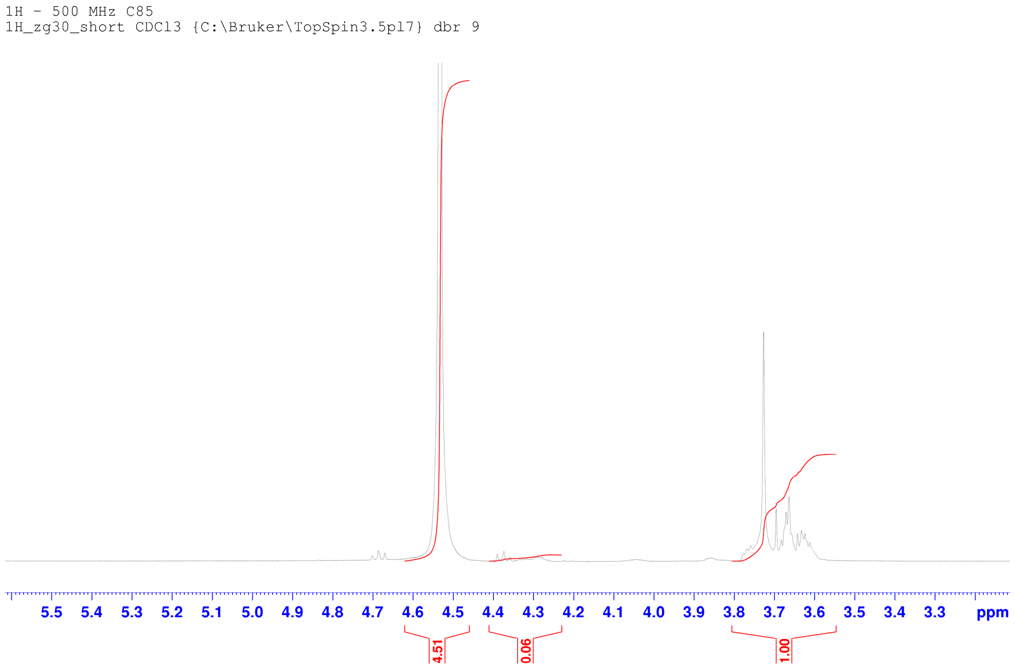


**Figure S22**. $^{1}$H-NMR spectrum in trichloromethane D1 for catalyst phosphate at a reaction temperature of 140°C. For reaction conditions see **Table S4**.

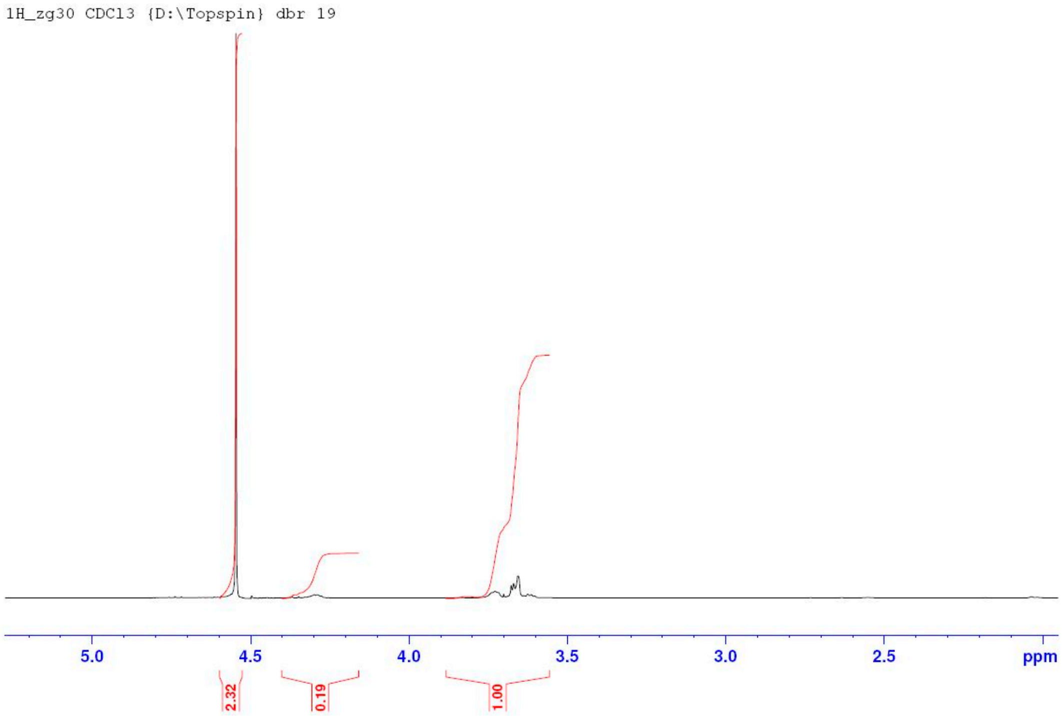


**Figure S23.** $^{1}$H-NMR spectrum in trichloromethane D1 for catalyst phosphate at a reaction temperature of 150°C. For reaction conditions see **Table S4**.

## 5.3 Vanadate

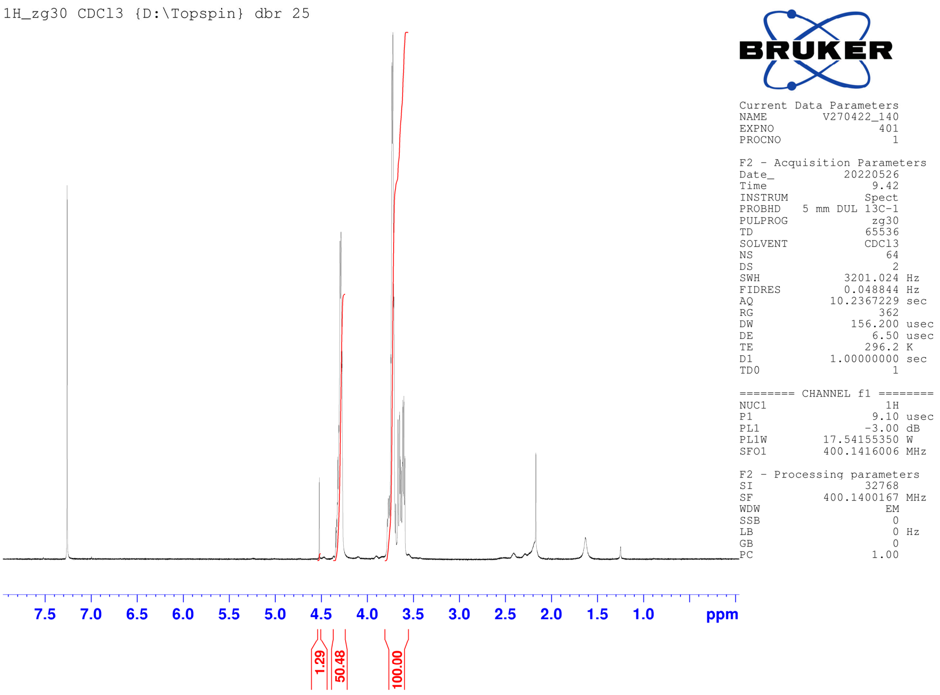


**Figure S24**. $^{1}$H-NMR spectrum in trichloromethane D1 for catalyst orthovanadate at a reaction temperature of 140°C. For reaction conditions see **Table S6**.

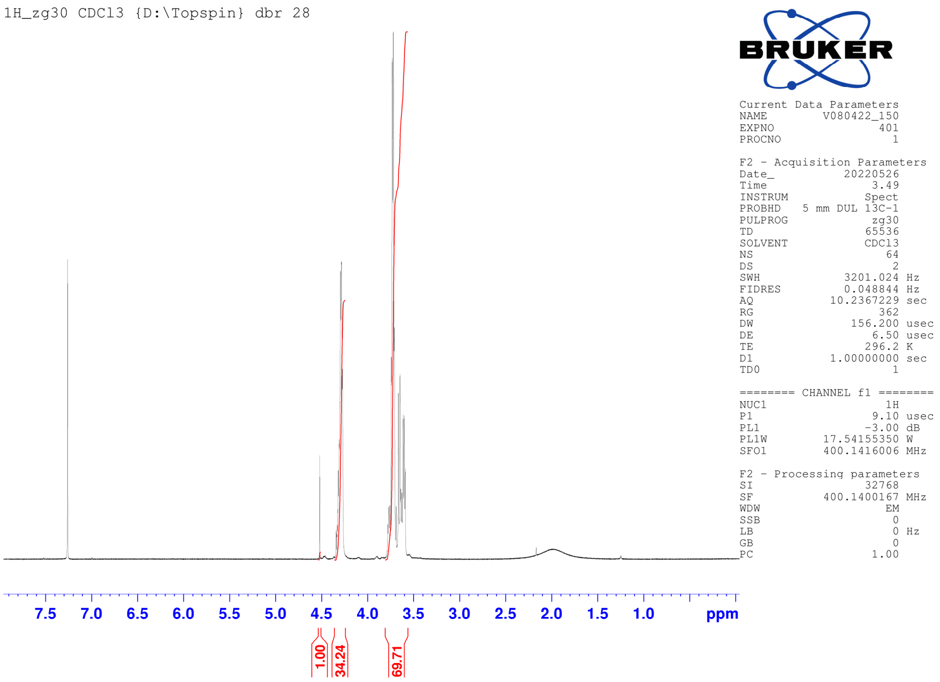


**Figure S25**. $^{1}$H-NMR spectrum in trichloromethane D1 for catalyst orthovanadate at a reaction temperature of 150°C. For reaction conditions see **Table S6**.

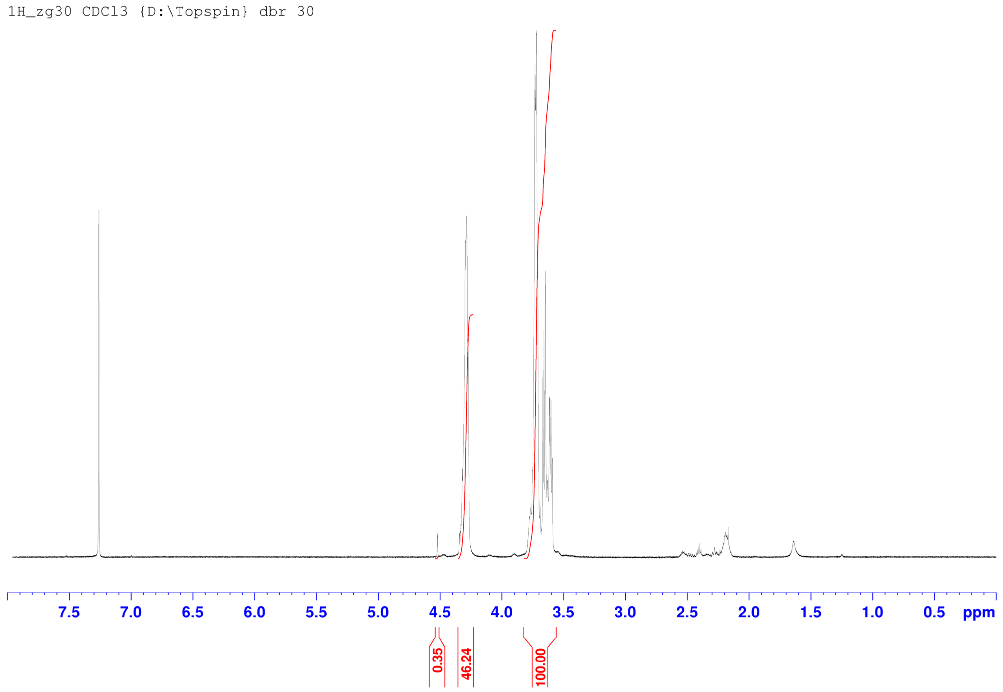


**Figure S26**. **1**H-NMR spectrum in trichloromethane D1 for catalyst orthovanadate at a reaction temperature of 160°C. For reaction conditions see **Table S6**.

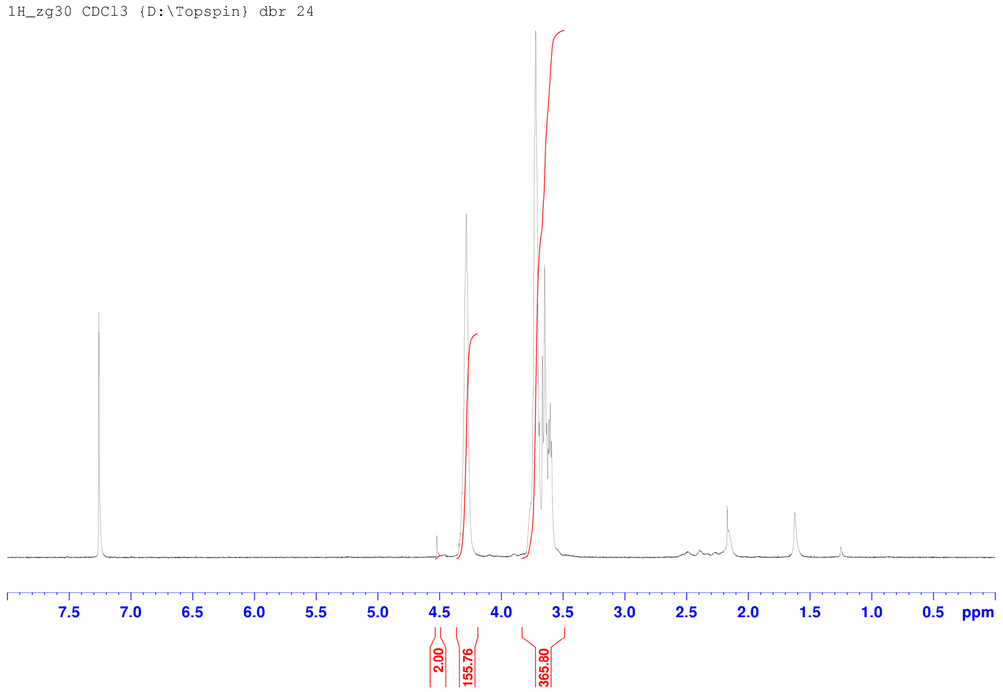


**Figure S27**. $^{1}$H-NMR spectrum in trichloromethane D1 for catalyst orthovanadate at a reaction temperature of 150°C. For reaction conditions see **Table S6**.

# 6 Additional Referenz Supporting information

[1] Hediger, H.J. Infrarotspektroskopie : Grundlagen, Anwendungen, Interpretation mit zahlreichen Tabellen und 32 Spektren. In Infrarotspektroskopie Grundlagen, Anwendungen, Interpret. Mit Zahlreichen Tabellen Und 32 Spektren, Akademische Verlagsgesellschaft, Frankfurt am Main, 1971: pp. 29–169, 173–195.

[2] H.J. Hediger, Infrarotspektroskopie Theorie : Grundlagen, Anwendungen, Interpretation mit zahlreichen Tabellen und 32 Spektren, in: Infrarotspektroskopie Grundlagen, Anwendungen, Interpret. Mit Zahlreichen Tabellen Und 32 Spektren, 11th ed., Frankfurt am Main, 1971: pp. 1–25.

[3] K. Cammann, Infrarotspektroskopie, in: Instrumentelle Anal. Chemie; Verfahren, Anwendungen, Qual., 1st ed., Spektrum Akademischer Verlag, Heidelberg - Berlin, 2001: pp. 5–24; 5–51.

[4] T. Fox, Hesse-Meier-Zeeh, Infrarot- und Ramanspektren, in: S. Bienz, L. Bigler, T. Fox, H. Meier (Eds.), Spektrosk. Methoden Der Org. Chemie, 9th ed., George Thieme Verlag, Stuttgart u. a., 2016: pp. 37–74.

[5] G. Schwedt, Infrarot- und Raman-Spektroskopie, in: Taschenatlas Der Anal., George Thieme Verlag, Stuttgart u. a., 1992: pp. 114–121.
